# Transcriptome profiling of developing leaf and shoot apices to reveal the molecular mechanism and co-expression genes responsible for the wheat heading date

**DOI:** 10.1186/s12864-021-07797-7

**Published:** 2021-06-23

**Authors:** Yuxin Yang, Xueying Zhang, Lifen Wu, Lichao Zhang, Guoxiang Liu, Chuan Xia, Xu Liu, Xiuying Kong

**Affiliations:** 1 The National Key Facility for Crop Gene Resources and Genetic Improvement, Institute of Crop Sciences, Chinese Academy of Agricultural Sciences, 100081 Beijing, China; 2 Hebei sub-center of National Maize Improvement Center of China, Key Laboratory of Crop Germplasm Resources of Northern China (Ministry of Education), College of Agronomy, Hebei Agricultural University, 071001 Baoding, China

**Keywords:** Wheat, Heading date, Gene expression, Transcription factors, Weighted gene co-expression network analysis

## Abstract

**Background:**

Wheat is one of the most widely planted crops worldwide. The heading date is important for wheat environmental adaptability, as it not only controls flowering time but also determines the yield component in terms of grain number per spike.

**Results:**

In this research, homozygous genotypes with early and late heading dates derived from backcrossed progeny were selected to conduct RNA-Seq analysis at the double ridge stage (W2.0) and androgynous primordium differentiation stage (W3.5) of the leaf and apical meristem, respectively. In total, 18,352 differentially expressed genes (DEGs) were identified, many of which are strongly associated with wheat heading date genes. Gene Ontology (GO) enrichment analysis revealed that carbohydrate metabolism, trehalose metabolic process, photosynthesis, and light reaction are closely related to the flowering time regulation pathway. Based on MapMan metabolic analysis, the DEGs are mainly involved in the light reaction, hormone signaling, lipid metabolism, secondary metabolism, and nucleotide synthesis. In addition, 1,225 DEGs were annotated to 45 transcription factor gene families, including LFY, SBP, and MADS-box transcription factors closely related to flowering time. Weighted gene co-expression network analysis (WGCNA) showed that 16, 336, 446, and 124 DEGs have biological connections with *Vrn1-5 A*, *Vrn3-7B*, *Ppd-1D*, and *WSOC1*, respectively. Furthermore, *TraesCS2D02G181400* encodes a MADS-MIKC transcription factor and is co-expressed with *Vrn1-5 A*, which indicates that this gene may be related to flowering time.

**Conclusions:**

RNA-Seq analysis provided transcriptome data for the wheat heading date at key flower development stages of double ridge (W2.0) and androgynous primordium differentiation (W3.5). Based on the DEGs identified, co-expression networks of key flowering time genes in *Vrn1-5 A*, *Vrn3-7B*, *WSOC1*, and *Ppd-1D* were established. Moreover, we discovered a potential candidate flowering time gene, *TraesCS2D02G181400*. Taken together, these results serve as a foundation for further study on the regulatory mechanism of the wheat heading date.

**Supplementary Information:**

The online version contains supplementary material available at 10.1186/s12864-021-07797-7.

## Background

Wheat (*Triticum aestivum* L.) is one of the most important and widely distributed food crops. The wide global planting area of bread wheat is attributed to its high adaptability to the natural environment. The heading date or flowering time is an important adaptive trait for crop genetic breeding.

Heading date regulation networks mainly involve vernalization, photoperiod, and hormones (such as gibberellin) [1, 2]. There are three important regulatory genes in the vernalization pathway: *Vernalization1* (*Vrn1-5 A*, *Vrn1-5B*, *Vrn1-5D*) [3–6], *Vernalization2* (*Vrn2*) [1, 4], and *Vernalization3* (*Vrn3*) [3–6]. *Vrn1* promotes flowering, with three homologous genes in wheat: *Vrn1-5 A*, *Vrn1-5B*, and *Vrn1-5D*. *Vrn-2* is the main flowering suppressor gene, and it is downregulated by vernalization and short-day treatment [1, 4, 7]. *Vrn-3* is a mobile signal protein that moves from the leaf to the apical meristem and induces flowering time [1, 8]. The wheat response to the photoperiod is mainly controlled by the *photoperiod* (*Ppd*) genetic locus, and genes participating in the photoperiod pathway mainly include *Ppd-A1*, *Ppd-B1*, and *Ppd-D1* [9, 10]. Previous studies report that *Ppd-1* plays a key role in the inflorescence morphological structure and young spike development process [11]. *Ppd-1* accelerates flowering by upregulating *Vrn3* expression under long-day conditions [12, 13]. Wheat *SOC1* (*WSOC1*) is a key regulatory gene in the gibberellin regulatory pathway and affects the heading date in polyploid wheat [14]; upregulation of *WSOC1* can accelerate spike development [15].

Flower development in higher plants mainly occurs in three stages: the floral induction phase, the floral primordia phase, and the floral organ development phase. Double ridge and androgynous primordium differentiation are key stages for flower induction and floral meristem development in wheat. At the double ridge stage (W1.5-W2.0), spikelet protuberance appears from the base of the growth cone between axils of bract primordium and determines the number of spikelets on each spike; at the differentiation stage of the androgynous primordium, the first floret primordium at the base of the spikelet begins to differentiate, and the stem begins to extend (W3.5) [16]. Thus, to explore the molecular mechanism underlying heading date, we performed RNA-Seq analysis of two wheat accessions with a significant difference in heading date at W2.0 and W3.5.

With the completion of hexaploid bread wheat reference genomes (IWGSC, 2018), the use of bioinformatics methods to discover flowering time genes and to examine molecular characterization has significantly accelerated the study of wheat flowering time. Indeed, more than nine hundred flowering time genes have been identified in wheat by BLAST searches and OrthoMCL clustering methods [17]. Moreover, researchers have discovered eighty-four SNPs that are highly related to spike numbers based on GWAS [18]. Using the bulked segregant RNA-Seq method, a large deletion in the first intron of *Vrn-B1* causing heading date variation was identified, and some flowering time GO terms were identified [19].

However, wheat is a hexaploid species and has a complex genome, which poses a challenge for discovering flowering time genes and revealing their genetic characteristics. In this study, homozygous genotypes with an early heading date (WHd) and late heading date (MHd) were selected for RNA-SEq. We aimed to investigate transcriptional regulation at the two key flower development stages of double ridge and androgynous primordium differentiation and identify the important flowering time genes using weighted gene co-expression network construction. We anticipate that transcriptome analysis will allow for exploring the molecular characteristics of wheat flowering time and promoting wheat improvement.

## Results

### Generation of RNA-Seq data

In this study, we constructed 24 RNA-Seq libraries for 8 treatments, namely, WHd-A2.0, MHd-A2.0, WHd-L2.0, MHd-L2.0, WHd-A3.5, MHd-A3.5, WHd-L3.5 and MHd-L3.5; each treatment included 3 biological repeats. A total of 40–59.4 million reads were obtained for each sample by high-throughput sequencing. The amount of data ranged from 6 to 8.9 Gb, the Q30 value exceeded 87 %, and the GC content distribution was 50–56 %. Sequencing alignment showed that 89.35–97.79 % of the reads could be mapped to IWGSC RefSeq v1.1 (Table S1). Principal component analysis (PCA) of the eight raw sequencing datasets clustered the samples into four groups according to genotype, which showed good repeatability between the samples for subsequent analysis (Fig. S1).

### Identification of differentially expressed genes

**Fig. 1:**
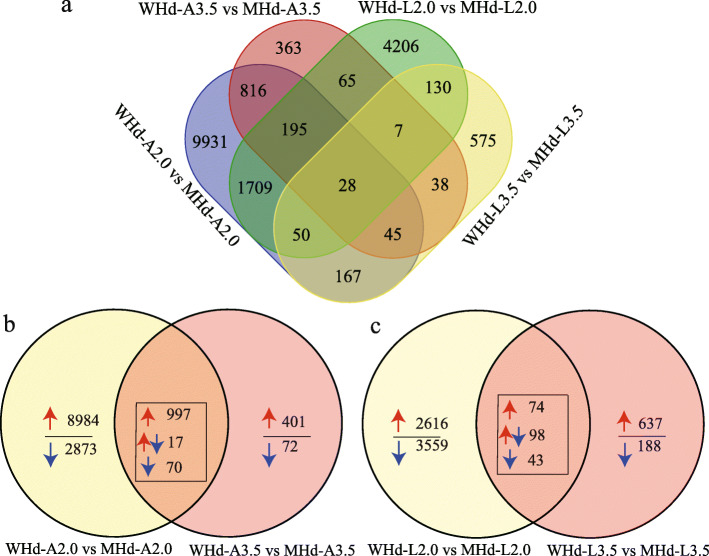
Venn diagram of all differentially expressed genes. **a** DEGs between WHd and MHd in different tissues and development periods. **b** Common DEGs in the apex of WHd vs. MHd between W2.0 and W3.5. **c** Common DEGs in leaves of WHd vs. MHd between W2.0 and W3.5

**Fig. 2:**
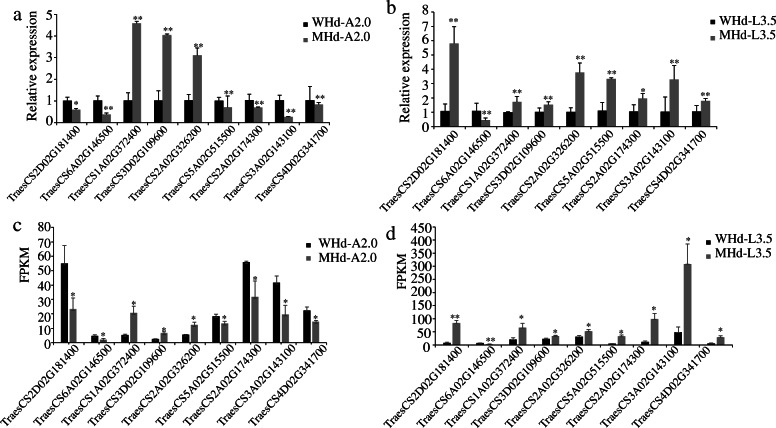
The gene expression level of differentially expressed genes measured using qRT-PCR. Each sample included three biological repeats. Three repeated biological experiments were carried out for each gene, and the error bar represents the SD of the means (*n* = 3). * represents *p* < 0.05, and ** represents *p* < 0.01. **a** The qRT-PCR results for DEGs at A2.0. **b** The qRT-PCR results for DEGs at L3.5. **c** The FPKM value for DEGs at A2.0. **d** The FPKM value for DEGs at L3.5

To identify genes that differed significantly between early and late heading genotypes during the development of young panicles, we analyzed the differentially expressed genes (DEGs) with strict quality control. The DEGs of L2.0, L3.5, A2.0, and A3.5 were compared between genotypes with an early heading time (WHd) and a late heading time (MHd). Finally, we identified 18,325 unique DEGs (Fig. 1a, Table S2), which are mainly distributed chromosome arm ends (Fig. S2). Specifically, 12,941 DEGs for the apical meristem at A2.0 were detected, 9,990 of which were upregulated and 2,951 downregulated (Fig. S3a); 1,557 DEGs at A3.5, 1,406 upregulated and 151 downregulated (Fig. S3b), 6,390 DEGs at L2.0, 2,735 upregulated and 3,655 downregulated (Fig. S3c), and 1,040 DEGs at L3.5, 764 upregulated and 276 downregulated (Fig. S3d), were also identified. The flowering time genes *Vrn1-5 A* [3], *Vrn3-7B* [5], *Ppd-1D* [9, 10], and *WSOC1* [14] were found to be differentially expressed. We also screened common differentially expressed genes at different developmental stages in the same tissue and observed that 1,084 genes were common DEGs at A2.0 and A3.5 (Fig. 1b), with 215 genes being common DEGs at L2.0 and L3.5 (Fig. 1c). We speculate that these genes may play important roles in spikelet development at the W2.0 and W3.5 stages. Moreover, nine genes from the above DEGs were selected for qRT-PCR analysis (Fig. 2a and b), and the FPKM values of the nine selected DEGs are shown in Table S7. Overall, the qRT-PCR expression trend of the nine DEGs agreed with the RNA-Seq data (Fig. 2c and d, Table S7).

### GO enrichment analysis of differentially expressed genes

**Fig. 3:**
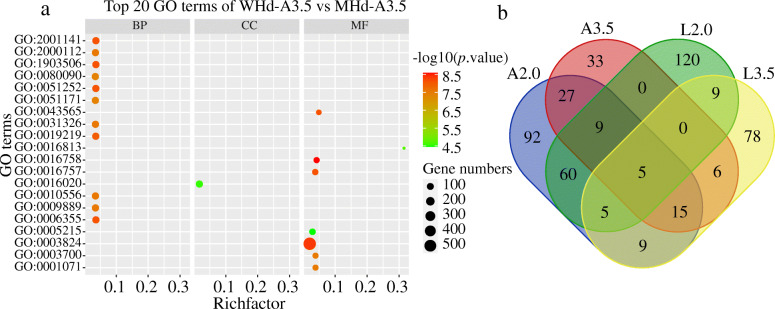
GO enrichment analysis of DEGs between WHd-A3.5 vs. MHd-A3.5. **a** Top 20 GO terms of WHd-A3.5 vs. MHd-A3.5. Each row corresponds to a significant GO term; columns represent the Richfactor (gene numbers of differentially expressed genes enriched in the pathway/all gene numbers in the background gene set). The bubble size represents gene numbers, and the color gradient represents the -log10 (*p-*value). **b** Venn plot showing the overlap of the number of significant GO regulatory pathways. A2.0: WHd-A2.0 vs. MHd-A2.0, A3.5: WHd-A3.5 vs. MHd-A3.5, L2.0: WHd-L2.0 vs. MHd-L2.0, L3.5: WHd-A3.5 vs. MHd-A3.5

To explore the regulatory pathways of these DEGs, GO enrichment analysis was conducted between early heading and late heading date wheat accessions. We identified 468 unique biological process (BP), molecular function (MF), and cellular component (CC) GO terms (Table S3, Fig. 3) (Fig. 3a; Fig. S4; Fig. S5; Fig. S6), with 222, 92, 208, and 127 significant GO terms in A2.0, A3.5, L2.0, and L3.5, respectively (Table S3). Interestingly, we discovered several flowering time-related GO terms, including GO:0005975 (carbohydrate metabolism) [20], GO:0005991 (trehalose metabolic process) [21], and GO:0019684 (photosynthesis, light reaction) [22]. Furthermore, 5 common GO terms, e.g., GO:0003700 (transcription factor activity, sequence-specific DNA binding), GO:0001071 (nucleic acid binding transcription factor activity), GO:0003824 (catalytic activity), GO:0043565 (sequence-specific DNA binding), and GO:0016491 (oxidoreductase activity), which were generally expressed in all comparative groups of DEGs (Fig. 3b), were detected.

### MapMan metabolic pathway analysis of differentially expressed genes

**Fig. 4:**
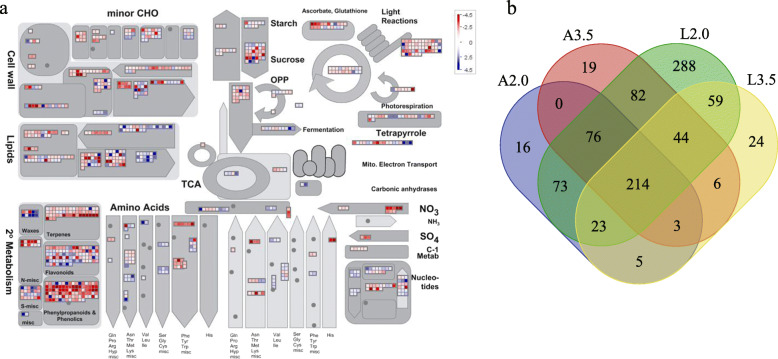
An overview of the metabolic pathways of differentially expressed genes between WHd-L2.0 vs. MHd-L2.0. **a** MapMan analysis of the DEGs between WHd-L2.0 vs. MHd-L2.0. Each inset presents a differentially expressed gene. The red lattice represents upregulated genes, and the blue lattice represents downregulated genes. The color scale presents the fold change value of DEGs. **b** Venn diagram showing the overlap of the number of significant MapMan metabolic regulatory pathways. A2.0: WHd-A2.0 vs. MHd-A2.0, A3.5: WHd-A3.5 vs. MHd-A3.5, L2.0: WHd-L2.0 vs. MHd-L2.0, L3.5: WHd-A3.5 vs. MHd-A3.5

The metabolic pathways of four comparative groups of DEGs were visualized by MapMan software (Fig. 4a; Fig. S7; Fig. S8; Fig. S9), and 932 unique metabolic pathways were ultimately identified (Table S4, Fig. 4b). The DEGs for A2.0 are mainly related to DNA-synthesis/chromatin structure (bin:28.1), signaling-receptor kinases (bin:30.2), signaling-light (bin:30.11), and hormone metabolism-jasmonate (bin:17.7). The DEGs for A3.5 were enriched in the category RNA-regulation of transcription-NAC domain transcription factor family (bin:27.3.27), PS-light-reaction-photosystem II (bin:1.1.1), hormone metabolism-ethylene-signal transduction (bin:17.5.2), protein-synthesis-initiation (bin:29.2.3). At L2.0, the metabolic pathway was mainly involved in secondary metabolism-phenylpropanoids-lignin biosynthesis (bin:16.2.1), and signaling (bin:30), RNA-regulation of transcription (bin:27.3.99). DEGs for L3.5 were enriched in DNA-synthesis/chromatin structure-histone (bin:28.1.3), transport (bin:34), metal handling-binding, chelation and storage (bin:15.2), and RNA-regulation of transcription and TCP transcription factor family (bin:27.3.29) categories. Moreover, 214 common metabolic pathways were screened, including bin:1.1.1 (light reaction), bin:11.8 (lipid metabolism), and bin:13.2.3.5 (amino acid metabolism) (Fig. 4b).

### Transcription factor classification and identification

To identify transcription factors (TFs) involved in the heading time development process, the DEGs were subjected to transcription factor analysis using iTAK software. According to the criteria of the plant transcription factor database (http://planttfdb.gao-lab.org/), a total of 1,225 transcription factors were classified into 45 transcription factor families (Table S5). A large number of DEGs are bHLH (129, 10.51 %), WRKY (109, 8.88 %), NAC (91, 7.41 %), AP2/ERF-ERF (88, 7.17 %), and MYB (64, 5.21 %) transcription factors (Table S5). Among them, we also identified several transcription factor families involved in the plant flowering process, including three LFY transcription factors [23], eighteen SBP transcription factors, thirty-six MADS-MIKC transcription factors and ten MADS-M-type transcription factors (Table S5).

**Fig. 5:**
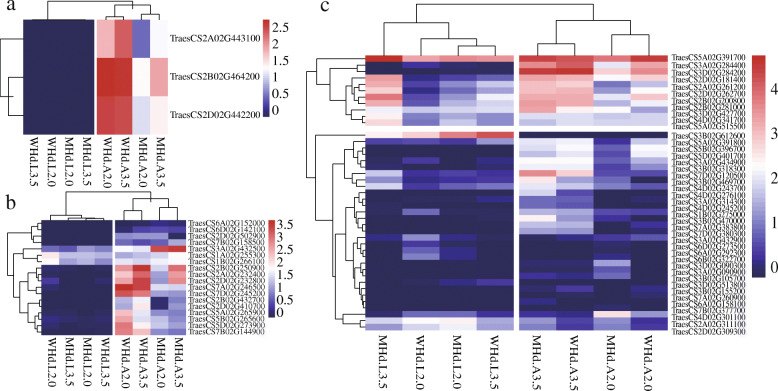
Gene expression levels of three transcription factor gene families. **a** The LFY transcription factor gene family. **b** The SBP transcription factor gene family. **c** The MADS-box transcription factor gene family

Gene expression level analysis showed that transcription factors are expressed at the critical period that determines flowering time. For example, we found three LFY transcription factor genes (*TraesCS2A02G443100*, *TraesCS2B02G464200*, *TraesCS2D02G442200*) to be highly expressed in the apical meristem, especially in WHd-A3.5 and WHd-A2.0 (Fig. 5a). Most SBP transcription factor genes were highly expressed in WHd-A2.0 and WHd-A3.5 (Fig. 5b). For the MADS-box gene family, the *TraesCS5A02G391700* (*Vrn1-5 A*) gene always showed a high expression level in both wild and mutant wheat, *TraesCS3B02G612600* was highly expressed in leaf tissue, and two genes (*TraesCS3D02G284200*, *TraesCS3A02G284400*) were highly expressed in the apical meristem (Fig. 5c).

### Construction of the flowering gene regulatory network

**Fig. 6:**
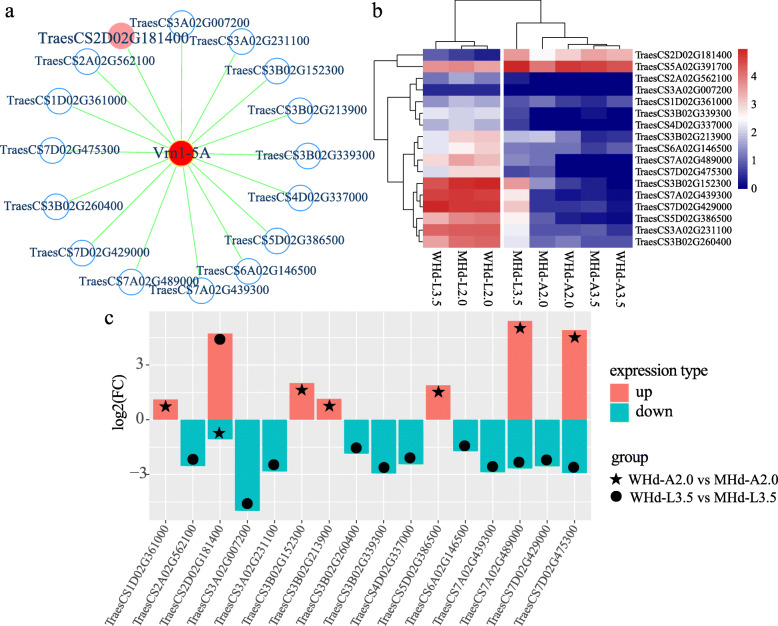
Construction of the flowering time regulatory network and expression level of differential genes. The red nodes in the network indicate high-confidence genes involved in the heading date. **a** The *Vrn1-5 A* flowering time regulatory network. **b** The expression heatmap of differentially expressed genes co-expressed with *Vrn1-5 A.* **c** The expression pattern of differentially expressed genes co-expressed with *Vrn1-5 A*. The red filled columns represent upregulated genes, and the blue filled columns represent downregulated genes

A total of 18,352 DEGs were used to construct a co-expression network. We calculated the average gene connectivity under different soft-thresholding powers and found that when β = 14, the co-expression network had scale-free characteristics (Fig. S10a; b; c; d). Finally, 17 co-expression modules were obtained by the “dynamic tree cut” method. Each branch in the cluster tree represents a gene set, and different modules are distinguished by different colors (Fig. S10e). The correlation value between the co-expression modules and samples was also calculated (Fig. S11). The reported flowering time genes *Vrn1-5 A* (*TraesCS5A02G391700*), *Vrn3-7B* (*TraesCS7B02G013100*), *Ppd-1D* (*TraesCS2D02G079600*), and *WSOC1* (*TraesCS4D02G341700*) were selected to build a gene co-expression network, and genes connected to the reported flowering time genes were extracted from the co-expression modules. We detected 16, 336, 446 and 124 DEGs with biological connections to *Vrn1-5 A* (Fig. 6a), *Vrn3-7B* (Fig. S12a), *Ppd-1D* (Fig. S13a), and *WSOC1* (Fig. S14a), respectively. The complete gene list related to the reported flowering time genes is summarized in Table S6.

### Analysis of *Vrn1-5 A*, *Vrn3-7B*, *Ppd-1D*, and *WSOC1* co-expression patterns

To further study the gene expression pattern of DEGs co-expressed with wheat heading date genes (*Vrn1-5 A*, *Vrn3-7B*, *Ppd-1D*, *WSOC1*), 16 genes co-expressed with *Vrn1-5 A* were divided into three patterns according to their gene expression level. Among them, *TraesCS2D02G181400* had the highest weight value (weight value = 0.18) with *Vrn1-5 A*. *TraesCS2D02G181400* is a MADS-MIKC transcription factor highly expressed in WHd-A3.5, MHd-A3.5, MHd-L3.5, and WHd-A2.0 but expressed at low levels in WHd-L2.0, MHd-L2.0, MHd-A2.0, and WHd-L3.5 (Fig. 6b). *TraesCS2D02G181400* was upregulated between WHd-A2.0 and MHd-A2.0 and downregulated between WHd-L3.5 and MHd-L3.5 (Fig. 6c). Five genes (*TraesCS1D02G339300*, *TraesCS2A02G562100*, *TraesCS3A02G007200*, *TraesCS3B02G361000* and *TraesCS4D02G337000*) were expressed at low levels at all stages in both the early and late heading date accessions (Fig. 6b). The remaining ten differentially expressed genes (*TraesCS3A02G231100*, *TraesCS3B02G152300*, *TraesCS3B02G213900*, *TraesCS3B02G260400*, *TraesCS5D02G386500*, *TraesCS6A02G146500*, *TraesCS7A02G439300*, *TraesCS7A02G489000*, *TraesCS7D02G429000* and *TraesCS7D02G475300*) showed higher expression levels in WHd-L2.0, WHd-L3.5, and MHd-L2.0 but lower expression in WHd-A2.0, WHd-A3.5, MHd-A2.0, MHd-A3.5, and MHd-L3.5 (Fig. 6b). The genes highly related to *Vrn3-7B* were mainly expressed in the leaf tissues (Fig. S12b). Genes associated with *Ppd-1D* tended to be expressed in the leaf (Fig. S13b). Some genes that were co-expressed with *WSOC1* were preferentially expressed in leaf tissues (Fig. S14b). Furthermore, by gene function annotation, we found some vital candidate flowering time genes in the co-expression network, such as *TraesCS1A02G220300*, *TraesCS2D02G181400*, *TraesCS3A02G143100* (Fig. 2), and *TraesCS5A02G166100* (Table 1). The detailed weight values of the co-expressed genes are given in Table S6.

**Table 1:** Functional annotation of genes co-expressed with flowering genes

Flowering gene	Candidate flowering time genes	Position in IWGSC1.1/Mb	Function annotation	Log2(FC)	*p*-value
*Vrn1-5A*	*TraesCS2D02G181400*	2D: 126.124 ~ 126.149	MADS-box transcription factor	4.72	3.90 × 10^-18^
*Vrn3-7B*	*TraesCS5A02G166100*	5A: 355.071 ~ 355.072	CONSTANS-like protein	3.66	8.66 × 10^-5^
	*TraesCS2D02G147100*	2D: 88.087 ~ 88.089	Gibberellin receptor GID1A	3.88	3.05 × 10^-5^
	*TraesCS5D02G170700*	5D: 267.762 ~ 267.764	CONSTANS-like protein	4.16	3.56 × 10^-5^
*Ppd-1D*	*TraesCS1A02G220300*	1A: 389.129 ~ 389.131	CONSTANS-like protein	-2.39	6.10 × 10^-4^
	*TraesCS1B02G326800*	1B: 553.024 ~ 553.025	Early light-induced protein	-3.15	1.27 × 10^-5^
	*TraesCS1B02G326900*	1B: 553.135 ~ 553.136	Early light-induced protein	-7.17	1.02 × 10^-12^
	*TraesCS1A02G314800*	1A: 506.473 ~ 506.476	Early light-induced protein	-4.09	3.21 × 10^-3^
*WSOC1*	*TraesCS3A02G143100*	3A:124.172 ~ 124.176	Flowering locus T	2.74	1.69 × 10^-7^
	*TraesCS2B02G200800*	2B: 180.040 ~ 180.060	MADS-box transcription factor	2.43	8.77 × 10^-7^
	*TraesCS3D02G144500*	3D: 132.976 ~ 132.977	Flowering locus T	2.28	2.95 × 10^-4^
	*TraesCS3B02G162000*	3B: 158.715 ~ 158.719	Flowering locus T	1.13	8.54 × 10^-4^
	*TraesCS5A02G515500*	5A: 747.807 ~ 757.809	MADS-box transcription factor	1.39	8.45 × 10^-4^
	*TraesCS3D02G144500*	3D: 132.976 ~ 132.977	Flowering locus T	2.28	2.95 × 10^-4^

## Discussion

Flowering time is an important agronomic trait that is regulated by many genes. The double ridge stage (W2.0) and androgynous primordium differentiation stage (W3.0) are vital during flower development. However, there are few studies on transcriptome analysis of heading date at W2.0 and W3.5 and few studies on co-expression networks construction of flowering time genes in wheat. In our study, we not only found known key heading date genes to be differentially expressed, such as *Vrn1-5 A*, *Vrn3-7B*, *Ppd-1D*, and *WSOC1* [3, 9, 14], but we also identified new DEGs involved in the process.

For example, GO:0005975 (carbohydrate metabolism) was enriched at A2.0 and L2.0, and GO:0005991 (trehalose metabolic process) was enriched at A2.0 and L3.5. A previous study reported that carbohydrates function in the regulation of plant flowering. For example, trehalose-6-phosphate (t6p) is suggested to be an indicator for measuring the carbohydrate status in plants [20, 21], and GO:0019684 (photosynthesis, light reaction), which is important for photoperiod pathways [22], is enriched only at L3.5. Gene function annotation revealed that *TraesCS1A02G339300* and *TraesCS1B02G351600* both encode trehalose-6-phosphate (t6p) (Table S2), and through MapMan metabolic pathway analysis, we discovered that they are involved in trehalose metabolism (bin:3.2.1). The t6p is an important endogenous signal that can influence the flowering time of *Arabidopsis thaliana* in both apical shoot and leaves [20]; thus, we speculate that *TraesCS1A02G339300* and *TraesCS1B02G351600* can be used to reveal the molecular mechanism by which of t6p affects flowering in wheat. Furthermore, functional annotation showed that *TraesCS3A02G400000* is a pfkB-like carbohydrate kinase family protein (Table S2); according to MapMan analysis, it is related to the major CHO metabolism-degradation-sucrose-fructokinase (bin:2.2.1.1) category.

Transcription factors play important roles in the regulation of plant flowering stage development. Our results showed that LFY, SBP, and MADS-box genes tended to be highly expressed in the apical meristem of wild type wheat (WHd-A2.0, WHd-A3.5). For example, *TraesCS2A02G443100* is an LFY transcription factor. The homologous gene in rice is *Os04g0598300*, which mainly controls the heading date and determines the development of branches and tillers in the ear, and overexpression of *Os04g0598300* can promote the heading date in rice [24]. In addition, the SBP transcription factor, *TraesCS7A02G246500*, was highly expressed at the WHd-A2.0 and WHd-A3.5 stages; its ortholog gene in *Arabidopsis* is *ATSPL9* (*AT2G42200*). Previous research showed that *ATSPL9* can regulate flowering time by promoting the transcription of MADS-box genes, including *FUL* (FRUITFULL), *SOC1* (SUPPRESSOR OF OVEREXPRESSION OF CONSTANS 1), and *AGL42* (AGAMOUS-LIKE) [25, 26]. *TraesCS3A02G284400* is a MIKC-type MADS-box gene. Its orthologous gene in rice *OsMADS32* is uniquely expressed in the early stage of the inflorescence meristem, e.g., spikelet primordium, maintaining flower organ characteristics and regulating flower development of rice flowers [27, 28]. Furthermore, we discovered that some MADS-type transcription factors were highly expressed in the leaves of both wildtype and mutant wheat, such as *Vrn1-5 A* (*TraesCS5A02G391700*), which indicates that this type of transcription factor can regulate flowering through different tissues, such as the leaf and apical meristem.

Moreover, many TFs, including WRKY, bZIP, bHLH, HSF, and MADS-box transcription factors, were involved in the flowering time gene co-expression network, was constructed by WGCNA (Table S6). In the *Vrn1-5 A* co-expression network, the MADS-MIKC transcription factor gene *TraesCS2D02G181400* was differentially expressed in A2.0 and L3.5 (Fig. 2a and b); its homolog in rice is *OsMADS18*. It is an AP1/FUL-like MADS-box gene that determines the formation of inflorescence meristem characteristics by interacting with PAP2 in the floral meristem [29]. We also discovered one basic helix-loop-helix (bHLH) transcription factor (*TraesCS5D02G386500*). Its homologous gene in *Arabidopsis* is *AT2G43010*, which negatively regulates the red light response mediated by phyB. Thus, we speculate that red light might affect expression of *Vrn1-5 A*. In addition, *TraesCS6A02G146500* (Fig. 2) encodes glucose-6-phosphate 1-dehydrogenase (G6DPH), providing energy and other metabolites for plant growth and development; we suggest that expression of vernalization genes requires other genes to provide energy. The *Vrn3-7B* gene co-expression network (Fig. S12a) also included four bZIP transcription factors (Table S6). Two CONSTANS-like proteins (*TraesCS5A02G166100*, *TraesCS5D02G170700*) were also discovered. The homolog of both *TraesCS5A02G166100* and *TraesCS5D02G170700* in *Arabidopsis* is *AT5G15840*, a zinc finger transcription factor-like protein that acts upstream of *FT* and *SOC1* and is mainly involved in flowering regulation under long-day conditions [30]. In the *Ppd-1D* co-expression network (Fig. S13a), identified transcription factors mainly comprised WRKY, bHLH, and HSF. We also identified one CONSTANS-like protein gene, *TraesCS1A02G220300*; its homolog gene in *Arabidopsis* is *AT5G57660*, which is involved in the flowering development process [31]. Some WRKY and MADS-box transcription factors were also identified in the *WSOC1* co-expression network (Fig. S14a). We detected three flowering locus T genes (*TraesCS3A02G143100*, *TraesCS3B02G162000*, *TraesCS3D02G144500*), which is consistent with previous reports noting that gibberellin can activate expression of Flowering Locus T [14]. Based on the above analysis, we suggest that the newly identified genes (*TraesCS2D02G181400*, *TraesCS5D02G386500*, *TraesCS6A02G146500*, *TraesCS5A02G166100*, *TraesCS5D02G170700*, *TraesCS1A02G220300*) associated with reported flowering time genes (*Vrn1-5 A*, *Vrn3-7B*, *Ppd-1D*, *WSOC1*) may play a key role in the wheat heading date regulatory pathway. Further research is warranted to reveal their biological functions.

## Conclusions

In this study, two key flower development stages were selected to conduct RNA-Seq analysis. Based on DEG analysis, many transcription factors co-expressed with key flowering time genes (*Vrn1-5 A*, *Vrn3-7B*, *Ppd-1D* and *WSOC1*) were identified by WGCNA. Subsequently, a potential flowering time regulatory gene, *TraesCS2D02G181400*, was discovered from the *Vrn1-5 A* network. The enriched transcriptome data at W2.0 and W3.5 will serve as a resource for elucidating the mechanism of wheat heading date regulation.

## Methods

### Plant materials

*m605* is a late heading mutant that was identified from the ethyl methanesulfonate mutant library of YZ4110. Previous studies conducted in our laboratory identified *m605* as a late heading mutant compared with its wild type parent YZ4110 [32]. To reveal the flowering time regulation model of genes for the heading date, homozygotes for the early heading date (WHd) and late heading date phenotype (MHd) (Fig. S15) derived from the progeny of BC_3_F_4_ were selected to conduct RNA-SEq. The purpose of using the backcrossed segregating population was to reduce false positive problems caused by the genetic background. Seeds of each wheat accession were planted in flowerpots and grown under 16 h light/22℃ and 8 h dark/18℃ (a long-day environment) in a culture room. The plants were transferred to a 4℃ environment for vernalization treatment for 4 weeks, where they developed to the one-leaf stage and were then cultured in a greenhouse until they were sampled. A total of twenty-four wheat samples were selected, including the apex of the wild/mutant type at the W2.0/W3.5 stage (WHd-A2.0, WHd-A3.5, MHd-A2.0, MHd-A3.5) and leaves of the wild/mutant type at the W2.0/W3.5 stage (WHd-L2.0, WHd-L3.5, MHd-L2.0, MHd-L3.0). All of the plant materials used in our study were derived from the Institute of Crop Sciences, Chinese Academy of Agricultural Sciences.

### RNA extraction, library construction, sequencing and quality control

The sampling period included the double ridge stage (W2.0) and androgynous primordium differentiation stage (W3.5) (Fig. S1), and the sampling position included the latest unfolding leaf and growth cone of the main stem. We used clean, liquid nitrogen-frozen forceps to quickly clamp the growth cone and place it in an RNase-free centrifuge tube (the centrifuge tube was first filled with liquid nitrogen). Newly unfolded leaves of the main stem corresponding to the growth cone were mixed at the same time; the sampling position was 2 cm away from the tip of the leaf, and a cut was made 4 cm away from the tip of the leaf to obtain a tissue sample with a length of 2 cm. All samples were rapidly frozen in liquid nitrogen and stored in a -80 °C environment. The TRIzol method was used to extract RNA from each sample; quality of RNA was detected by 1 % agarose gel electrophoresis, and the concentration of RNA was assessed using a Nanodrop 2000 instrument. High-quality RNA samples were sent to the ONMATH company (www.onmath.cn) to construct the sequencing library following Illumina’s standard pipeline. The constructed sequence libraries were sequenced with the Illumina HiSeq™ 4000 platform for 150 bp paired sequencing, and raw data were saved as fastq files. To remove the adaptors and lower quality reads at the head and end, we used Trimmomatic (version 0.35) [33] to conduct quality control of sequencing data with the following parameters: java -jar trimmomatic − 0.35.jar PE -threads 20 forward.fastq reverse.fastq forward_paired.fastq forward_unpaired.fastq reverse_paired.fastq reverse_unpaired.fastq ILLUMINACLIP:1.adapter.list:2:30:10 LEADING:10 TRAILING:10 SLIDINGWINDOW:1:10 MINLEN:50. FaQCs software (version 2.08) [34] was used to calculate the quality score, Q30 distribution, and GC distribution with the default parameters: FaQCs − 1 R1.clean.fastq − 2 R2.clean.fastq -d. --qc_only. PCA of RNA-seq data was carried out by OmicShare tools (http://www.omicshare.com/tools).

### Gene expression analysis of differentially expressed genes

RNA-Seq analysis of the sequencing libraries was performed according to the method reported by Perter et al. [35]. (1) Hisat2 software (version 2.5.3a) was used to construct an index of the reference genome of hexaploid wheat Chines Spring (IWGSC RefSeq v1.1, http://www.wheatgenome.org/Tools-and-Resources) with default parameters. (2) Clean reads were mapped to IWGSC RefSeq v1.1 by hisat2 with the parameters “hisat2 -x reference.genome.index -p 8 -X 400 --no-unal --dta − 1 input.R1.clean.fastq.gz -2 input.R2.clean.fastq.gz -S input.sam”, and the mapping results of reads were stored in a bam file. (3) Stringtie software (version 1.3.3b) [36] was used to carry out transcript assembly of alignments with the following parameters: stringtie -e -p 16 -B -G reference.genome.gff3 input.bam -o input.gtf. (4) The R package Ballgown [35] was employed to calculate gene expression levels, and we used FPKM (fragments per kilobase of transcript per million mapped reads) to measure gene expression values at the whole-genome level. In addition, we used HTseq-count software (version 0.11.1) [37] to count the read number of each bam file with the following command line: htseq-count --format bam --order pos --mode union --stranded no --type exon input.bam reference.genome.gtf > reference.counts.txt. The count files of reads were used to calculate differentially expressed genes with the R package egdeR [38]. The evaluation criteria for differentially expressed genes were an absolute log2 (fold change) greater than 1 and a false discovery rate (FDR) less than 0.05 [39, 40]. The distribution of DEGs along the three wheat subgenomes was determined by chromPlot software [41].

### GO enrichment analysis of DEGs

DEGs were submitted to the agrigo (http://systemsbiology.cau.edu.cn/agriGOv2/index.php) analysis toolkit [42, 43] for GO enrichment analysis. Annotation information provided by the IWGSC reference genome (IWGSC RefSeq v1.1) was used as a reference database. We used the singular enrichment analysis method through Fisher’s exact and Bonferroni multiple tests to screen significant GO terms.

### MapMan metabolic analysis of DEGs

The differential expression spectrum was mapped to the metabolic regulatory pathway in detail using MapMan (version 3.5.1) [44]. Considering that MapMan software lacks the mapping file from the Chinese Spring reference genome, we first extracted the coding sequence of DEGs and then uploaded this sequence to Mercator (http://www.plabipd.de/portal/mercator-sequence-annotation) for gene annotation and to obtain the corresponding mapping file. Then, the mapping file containing the gene expression value was imported into MapMan to analyze metabolic regulation [45, 46].

### Transcription factor analysis of DEGs

To explore the transcription factor changes during wheat heading date development, we extracted the protein sequences of DEGs from IWGSC RefSeq v1.1 and used the iTAK [47] pipeline (http://bioinfo.bti.cornell.edu/tool/itak) for transcriptome factor prediction and functional classification.

### Weighted gene co-expression network analysis and flowering time regulatory network construction

The R package WGCNA (version 1.70-3) [48, 49] was utilized to construct the flowering time co-expression network and identify candidate flowering time genes. The weight value is a parameter for measuring the co-expression value, which correlates positively correlated with the degree of co-expression. First, the “goodSamplesGenes” function was used to check the quality of the gene expression data, and the “pickSoftThreshold” function was applied to determine the soft power. Second, we calculated the “adjacency matrix” and “topological overlap matrix” according to the soft power value. Third, the initial gene expression matrix was obtained by hierarchical clustering of the dissimilarity matrix with the “hclust” function with the default parameters: minModuleSize: 30, networkType: “signed”, TOMType: “unsigned”, mergeCutHeight: 0.25. Based on the connectivity between co-expressed genes, Cytoscape software (version 3.7.2) [50] was used to visualize the flowering-related gene regulation network.

### Quantitative Real-Time PCR

We used Primer Premier 5 software to design gene-specific primers based on gene sequence. Each experiment included three biological repeats. We selected *GAPDH* (GenBank: AF251217.1) as the internal reference gene to correct gene expression. The PCR system included a 20 µL reaction volume, which consisted of 10 µL of SYBR Mix, 2 µL of cDNA, 0.8 µL of forward and reverse primers, and 7.2 µL of ddH_2_O. RT-PCR amplification in the Roche LightCycler 480 Real-Time System (Roche, Switzerland) platform was performed. The PCR amplification steps were 95 ℃ for 60 s, followed by 40 cycles at 95℃ for 5 s, 60℃ for 30 s, and 95℃ for 15 s. The 2(-delta delta C(T)) method was used for the relative gene expression analysis of DEGs [51]. All primer sequences used in this study are shown in Table S7.

## Supplementary Information

**Additional file 1: Figure S1.** PCA showing the inherent biological variation among different samples; the results confirmed that the samples can be classified into different categories.

**Additional file 2: Figure S2.** The distribution of differentially expressed genes along the three wheat subgenomes.

**Additional file 3: Figure S3.** Volcano map of DEGs between WHd and MHd at different developmental stages and in different tissues. The abscissa represents the log2 (FC) value of each gene, and the ordinate corresponds to adjusted *p*-values. Green dots represent downregulated genes, red dots represent upregulated genes, and dark dots represent genes not differentially expressed. (a) Volcano map of DEGs between WHd-A2.0 vs MHd-A2.0. (b) Volcano map of DEGs between WHd-A3.5 vs MHd-A3.5. (c) Volcano map of DEGs between WHd-L2.0 vs MHd-L2.0. (d) Volcano map of DEGs between WHd-L3.5 vs MHd-L3.5.

**Additional file 4: Figure S4.** GO enrichment analysis of DEGs between WHd-A2.0 vs MHd-A2.0.

**Additional file 5: Figure S5. **GO enrichment analysis of DEGs between WHd-L2.0 vs MHd.L2.0.

**Additional file 6: Figure S6.** GO enrichment analysis of DEGs between WHd-L3.5 vs MHd-L3.5.

**Additional file 7: Figure S7.** Overview of the metabolic pathway of differentially expressed genes between WHd-A2.0 vs MHd-A2.0.

**Additional file 8: Figure S8.** Overview of the metabolic pathway of differentially expressed genes between WHd-A3.5 vs MHd-A3.5.

**Additional file 9: Figure S9.** Overview of the metabolic pathway of differentially expressed genes between WHd-L3.5 vs MHd-L3.5.

**Additional file 10: Figure S10.** Identification of the soft-threshold power in WGCNA. (a) Scale-free fit index analysis under different soft-thresholding powers (β). Ordinates represent the relationship between the degree of connection (k) and P(k). (b) Mean gene connectivity analysis under different soft-thresholding powers (β). (c) Histogram of connectivity distribution when β = 14. (d) A check of the scale-free topology when β = 14. (e) Dendrogram of all differentially expressed genes clustered based on a dissimilarity measure (1-TOM).

**Additional file 11: Figure S11.** Heatmap of module-trait relationships. Each row is a module eigenvalue that is represented by different colors, and each column represents a sample name. Correlation between sample names and module eigenvalues was calculated by the Pearson correlation coefficient. Red represents a high correlation and green represents a negative correlation between samples and module eigenvalues.

**Additional file 12: Figure S12.** Construction of the flowering time regulatory network and expression level of the differential genes. The red nodes in the network indicate high-confidence genes involved in the heading date. (a) The *Vrn3-7B* flowering time regulatory network. (b) The expression heatmap of differentially expressed genes that were co-expressed with *Vrn3-7B*.

**Additional file 13: Figure S13.** Construction of the flowering time regulatory network and expression level of differential genes. The red nodes in the network indicate high-confidence genes involved in the heading date. (a) The *Ppd1-1D* flowering time regulatory network. (b) The expression heatmap of differentially expressed genes co-expressed with *Ppd1-1D*.

**Additional file 14: Figure S14.** Construction of the flowering time regulatory network and expression level of the differential genes. The red nodes in the network indicate high-confidence genes involved in the heading date. (a) Representative *WSOC1* flowering time regulatory network. (b) The expression heatmap of differentially expressed genes that were co-expressed with *WSOC1*.

**Additional file 15: Figure S15.** The development stages of the shoot apex. A: Double ridge stage; B: Pistil and stamen primordium differentiation stage. Note: The red arrow indicates the characteristics of the development period.

**Additional file 16: Table S1.** Statistical data of each RNA-Seq sample, including read number, data size, Q30 value, GC content, and alignment results.

**Additional file 17: Table S2.** DEG information between WHd-A2.0 and MHd-A2.0, WHd-A3.5 and MHd-A3.5, WHd-L2.0 and MHd-L2.0, and WHd-L3.5 and MHd-L3.5.

**Additional file 18: Table S3.** GO enrichment analysis of DEGs between the four groups.

**Additional file 19: Table S4.** Detailed list of the MapMan annotations of the four groups of DEGs.

**Additional file 20: Table S5.** Identification of transcription factors using differentially expressed genes.

**Additional file 21: Table S6.** Functional annotation of genes co-expressed with flowering time genes.

**Additional file 22: Table S7. **Sheet1:Sequences of primers used in qRT-PCR experiments. Sheet2: Relative expression data for the nine DEGs. Sheet3: FPKM values for the nine DEGs.

## Data Availability

The datasets used and analyzed during the current study have been successfully stored in the SRA database of NCBI; the RNA-Seq accession number is PRJNA668815 (https://www.ncbi.nlm.nih.gov/bioproject/PRJNA668815). The reference genome and annotation files of hexaploid wheat Chines Spring (IWGSC RefSeq v1.1) were downloaded from the http://www.wheatgenome.org/Tools-and-Resources.

## Abbreviations

DEG: Differentially expressed gene
RNA-Seq: RNA sequencing
GO terms: Gene Ontology terms
WGCNA: Weighted gene co-expression network analysis
EMS: Ethyl methanesulfonate
PCA: Principal component analysis
FPKM: Fragment per kilobase of transcripts effective length per million fragments mapped to all transcripts
qRT-PCR: Quantitative real-time polymerase chain reaction

## References

[1] Distelfeld A, Li C, Dubcovsky J (2009). Regulation of flowering in temperate cereals. Curr Opin Plant Biol.

[2] Shi C, Zhao L, Zhang X, Lv G, Pan Y, Chen F (2019). Gene regulatory network and abundant genetic variation play critical roles in heading stage of polyploidy wheat. BMC Plant Biol.

[3] Yan L, Loukoianov A, Tranquilli G, Helguera M, Fahima T, Dubcovsky J (2003). Positional cloning of the wheat vernalization gene *VRN1*. Proc Natl Acad Sci U S A.

[4] Yan L, Loukoianov A, Blechl A, Tranquilli G, Ramakrishna W, SanMiguel P, Bennetzen JL, Echenique V, Dubcovsky J (2004). The wheat *VRN2* gene is a flowering repressor down-regulated by vernalization. Science.

[5] Yan L, Fu D, Li C, Tranquilli G, Bonafede M, Sanchez A, Valarik M, Yasuda S, Dubcovsky J (2006). The wheat and barley vernalization gene *VRN3* is an orthologue of FT. Proc Natl Acad Sci U S A.

[6] Kippes N, Zhu J, Chen A, Vanzetti L, Lukaszewski A, Nishida H, Kato K, Dvorak J, Dubcovsky J (2014). Fine mapping and epistatic interactions of the vernalization gene *VRN-D4* in hexaploid wheat. Mol Genet Genomics.

[7] Dubcovsky J, Loukoianov A, Fu D, Valarik M, Sanchez A, Yan L (2006). Effect of photoperiod on the regulation of wheat vernalization genes *VRN1* and *VRN2*. Plant Mol. Biol..

[8] Chen A, Dubcovsky J (2012). Wheat TILLING mutants show that the vernalization gene *VRN1* down-regulates the flowering repressor *VRN2* in leaves but is not essential for flowering. PLoS Genet.

[9] Turner A, Beales J, Faure S (2005). The pseudo-response regulator *Ppd-H1* provides adaptation to photoperiod in barley. Science.

[10] Beales J, Turner A, Griffiths S, Snape JW, Laurie DA (2007). A pseudo-response regulator is misexpressed in the photoperiod insensitive *Ppd-D1a* mutant of wheat (*Triticum aestivum* L.). Theor Appl Genet.

[11] Boden SA, Cavanagh C, Cullis BR, Ramm K, Greenwood J, Jean FE, Trevaskis B, Swain SM (2015). *Ppd-1* is a key regulator of inflorescence architecture and paired spikelet development in wheat. Nature Plants.

[12] Shaw LM, Turner AS, Laurie DA, Baliga NS, Wang JT, Ramage D, Amin N, Schwikowski B, Ideker T (2012). The impact of photoperiod insensitive *Ppd-1a* mutations on the photoperiod pathway across the three genomes of hexaploid wheat (*Triticum aestivum*). Plant J.

[13] Nishida H, Yoshida T, Kawakami K (2013). Structural variation in the 5′ upstream region of photoperiod insensitive alleles *Ppd-A1a* and *Ppd-B1a* identified in hexaploid wheat (*Triticum aestivum* L.) and their effect on heading time. Mol Breeding.

[14] Shitsukawa N, Ikari C, Mitsuya T (2007). Wheat SOC1 functions independently of WAP1/VRN1, an integrator of vernalization and photoperiod flowering promotion pathways. Physiol Plant.

[15] Shitsukawa N, Takagishi A, Ikari C, Takumi S, Murai K (2006). *WFL*, a wheat *FLORICAULA/LEAFY* ortholog, is associated with spikelet formation as lateral branch of the inflorescence meristem. Genes Genet Syst..

[16] Waddington SR, Cartwright PM, Wall PC (1983). A quantitative scale of spike initial and pistil development in barley and wheat. Ann Bot.

[17] Peng FY, Hu Z, Yang R (2015). Genome-Wide Comparative analysis of flowering-related genes in *Arabidopsis*, wheat, and barley. International Journal of Plant Genomics.

[18] Liu J, Xu Z, Fan X, Zhou Q, Cao J, Wang F, Ji G, Yang L, Feng B, Wang T (2018). A genome-wide association study of wheat spike related traits in China. Front Plant Sci.

[19] Li Y, Xiong H, Guo H, Zhou C, Xie Y, Zhao L, Gu J, Zhao S, Ding Y, Liu L (2020). Identification of the vernalization gene *VRN-B1* responsible for heading date variation by QTL mapping using a RIL population in wheat. BMC Plant Biol.

[20] Wahl V, Ponnu J, Schlereth A (2013). Regulation of flowering by trehalose-6-phosphate signaling in *Arabidopsis thaliana*. Science.

[21] Ponnu J, Wahl V, Schmid M (2011). Trehalose-6-phosphate: connecting plant metabolism and development. Front Plant Sci.

[22] Ananyev G, Gates C, Kaplan A, Dismukes GC (2017). Photosystem II-cyclic electron flow powers exceptional photoprotection and record growth in the microalga Chlorella ohadii. Biochim Biophys Acta Bioenerg.

[23] Goslin K, Zheng B, A SM, Rae L, Ryan PT, Kwaśniewska K, Thomson B, Ó’Maoiléidigh DS, Madueño F, Wellmer F, Graciet E (2017). Transcription factor interplay between LEAFY and APETALA1/CAULIFLOWER during floral initiation. Plant Physiol.

[24] Nagashree KL, Ahmed MF (2008). Electrocatalytic oxidation of methanol on Pt modified polyaniline in alkaline medium. Synth Met.

[25] Wu G, Poethig RS (2006). Temporal regulation of shoot development in *Arabidopsis thaliana* by miR156 and its target *SPL3*. Development.

[26] Yamaguchi A, Wu M, Yang L, Wu G, Poethig RS, Wagner D (2009). The microRNA-regulated SBP-Box transcription factor SPL3 is a direct upstream activator of LEAFY, FRUITFULL, and APETALA1. Dev Cell.

[27] Wang H, Zhang L, Cai Q, Hu Y, Jin Z, Zhao X, Fan W, Huang Q, Luo Z, Chen M (2015). OsMADS32 interacts with PI-like proteins and regulates rice flower development. J Integr Plant Biol.

[28] Sang X, Li Y, Luo Z, Ren D, Fang L, Wang N, Zhao F, Ling Y, Yang Z, Liu Y (2012). CHIMERIC FLORAL ORGANS1, encoding a monocot-specific MADS box protein, regulates floral organ identity in rice. Plant Physiol.

[29] Kobayashi K, Yasuno N, Sato Y, Yamazaki R, Kimizu M, Yoshida H, Nagamura Y, Kyozuka J (2012). Inflorescence meristem identity in rice is specified by overlapping functions of three *AP1/FUL*-like MADS box genes and *PAP2*, a *SEPALLATA* MADS box gene. Plant Cell.

[30] Yu Y, Qiao L, Chen J, Rong Y, Zhao Y, Cui X, Xu J, Hou X, Dong C (2020). Arabidopsis REM16 acts as a B3 domain transcription factor to promote flowering time via directly binding to the promoters of *SOC1* and *FT*. Plant J.

[31] Hassidim M, Harir Y, Yakir E, Kron I, Green RM (2009). Over-expression of *CONSTANS-LIKE 5* can induce flowering in short-day grown *Arabidopsis*. Planta.

[32] Zhang X, Liu G, Zhang L, Xia C, Zhao T, Jia J, Liu X, Kong X (2018). Fine mapping of a novel heading date gene, *TaHdm605*, in hexaploid wheat. Front Plant Sci..

[33] Bolger AM, Lohse M, Usadel B (2014). Trimmomatic: a flexible trimmer for Illumina sequence data. Bioinformatics.

[34] Lo CC, Chain PSG (2014). Rapid evaluation and quality control of next generation sequencing data with FaQCs. BMC Bioinformatics.

[35] Pertea M, Kim D, Pertea GM, Leek JT, Salzberg SL (2016). Transcript-level expression analysis of RNA-seq experiments with HISAT, StringTie and Ballgown. Nat Protoc.

[36] Pertea M, Pertea GM, Antonescu CM, Chang TC, Mendell JT, Salzberg SL (2015). StringTie enables improved reconstruction of a transcriptome from RNA-seq reads. Nat Biotechnol.

[37] Anders S, Pyl PT, Huber W (2015). HTSeq - a Python framework to work with high-throughput sequencing data. Bioinformatics.

[38] Robinson MD, McCarthy DJ, Smyth GK (2010). edgeR: a Bioconductor package for differential expression analysis of digital gene expression data. Bioinformatics.

[39] Audic S, Claverie JM (1997). The significance of digital gene expression profiles. Genome Res.

[40] Mariani TJ, Budhraja V, Mecham BH, Gu CC, Watson MA, Sadovsky Y (2003). A variable fold change threshold determines significance for expression microarrays. FASEB J.

[41] Oróstica KY, Verdugo RA (2016). chromPlot: visualization of genomic data in chromosomal context. Bioinformatics.

[42] Du Z, Zhou X, Ling Y, Zhang Z, Su Z (2010). agriGO: a GO analysis toolkit for the agricultural community. Nucleic Acids Res..

[43] Tian T, Liu Y, Yan H (2017). agriGO v2. 0: a GO analysis toolkit for the agricultural community, 2017 update. Nucleic Acids Res.

[44] Thimm O, Bläsing O, Gibon Y (2004). MAPMAN: a user-driven tool to display genomics data sets onto diagrams of metabolic pathways and other biological processes. Plant J..

[45] Klie S, Nikoloski Z (2012). The choice between MapMan and Gene Ontology for automated gene function prediction in plant science. Front Genet.

[46] Chandran AKN, Lee GS, Yoo YH, Yoon UH, Ahn BO, Yun DW, Kim JH, Choi HK, An G, Kim TH (2016). Functional classification of rice flanking sequence tagged genes using MapMan terms and global understanding on metabolic and regulatory pathways affected by dxr mutant having defects in light response. Rice.

[47] Zheng Y, Jiao C, Sun H, Rosli HG, Pombo MA, Zhang P, Banf M, Dai X, Martin GB, Giovannoni JJ (2016). iTAK: a program for genome-wide prediction and classification of plant transcription factors, transcriptional regulators, and protein kinases. Mol Plant.

[48] Zhang B, Horvath S. A general framework for weighted gene co-expression network analysis. Stat Appl Genet Mol Biol. 2005;4(1). 10.2202/1544-6115.1128. 10.2202/1544-6115.1128 16646834

[49] Langfelder P, Horvath S (2008). WGCNA: an R package for weighted correlation network analysis. BMC Bioinformatics.

[50] Shannon P, Markiel A, Ozier O (2003). Cytoscape: a software environment for integrated models of biomolecular interaction networks. Genome Res.

[51] Livak KJ, Schmittgen TD (2001). Analysis of relative gene expression data using real-time quantitative PCR and the 2^–∆∆C^_T_ method. Methods.

