# Supplementary figures and images for: Transcriptome profiling of developing leaf and shoot apices to reveal the molecular mechanism and co-expression genes responsible for the wheat heading date
Source: BMC Genomics. 2021 Jun 23;22:468. doi: 10.1186/s12864-021-07797-7 (PMC8220847; doi:10.1186/s12864-021-07797-7)

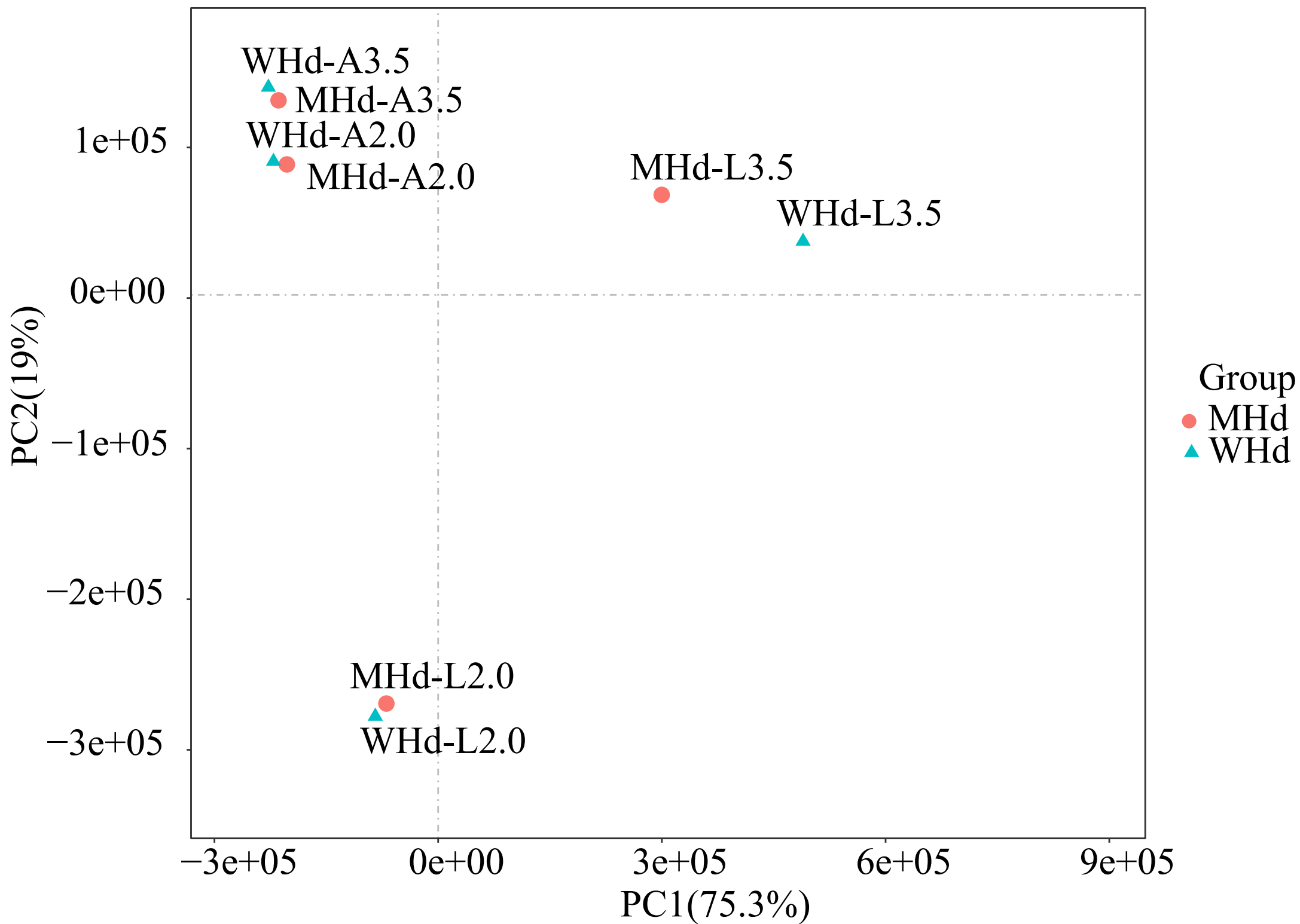

Supplement: Supplementary file 1 — Additional file 1: Figure S1. PCA showing the inherent biological variation among different samples; the results confirmed that the samples can be classified into different categories. [file 12864_2021_7797_MOESM1_ESM.pdf]

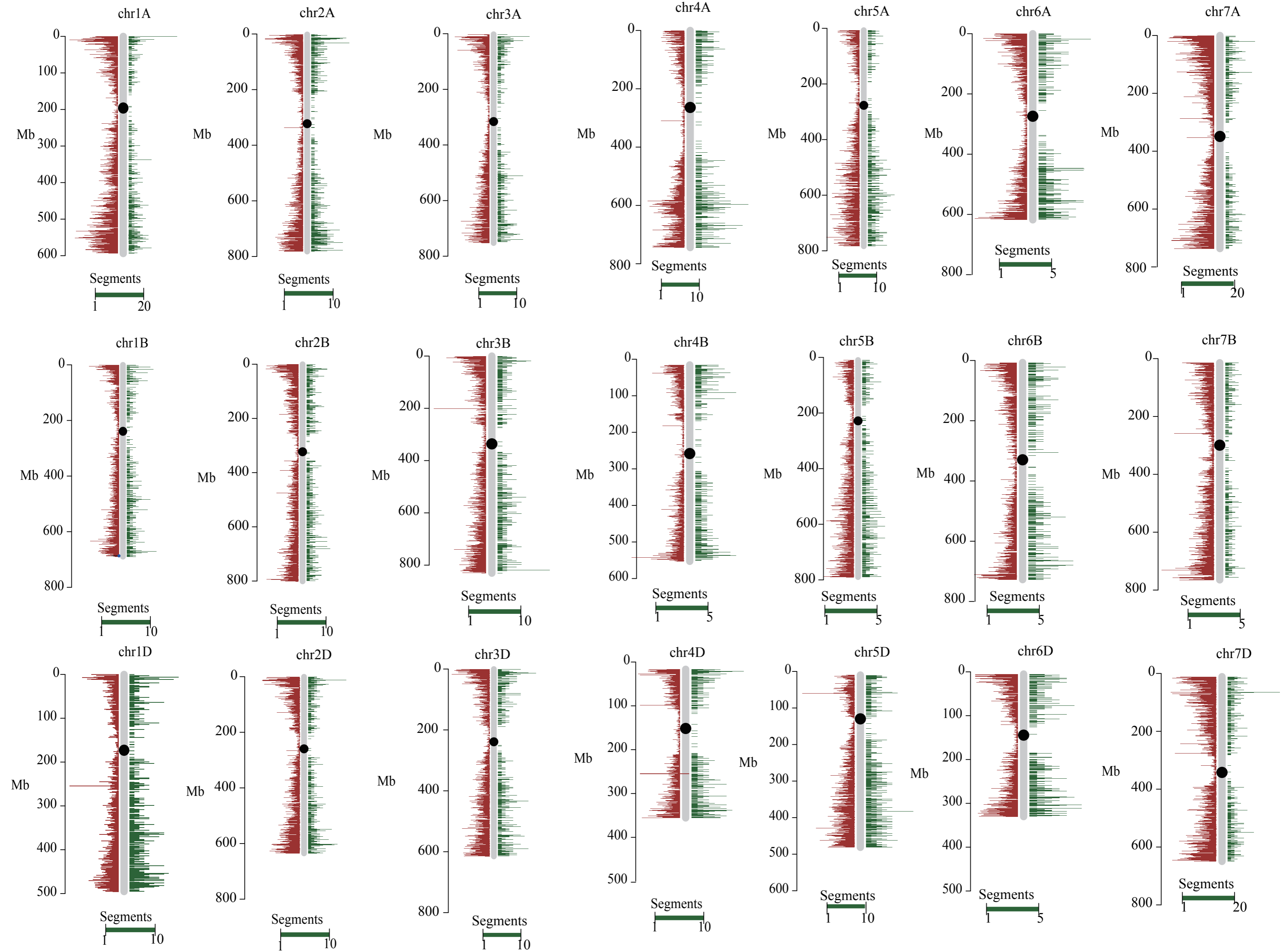

Supplement: Supplementary file 2 — Additional file 2: Figure S2. The distribution of differentially expressed genes along the three wheat subgenomes. [file 12864_2021_7797_MOESM2_ESM.pdf]

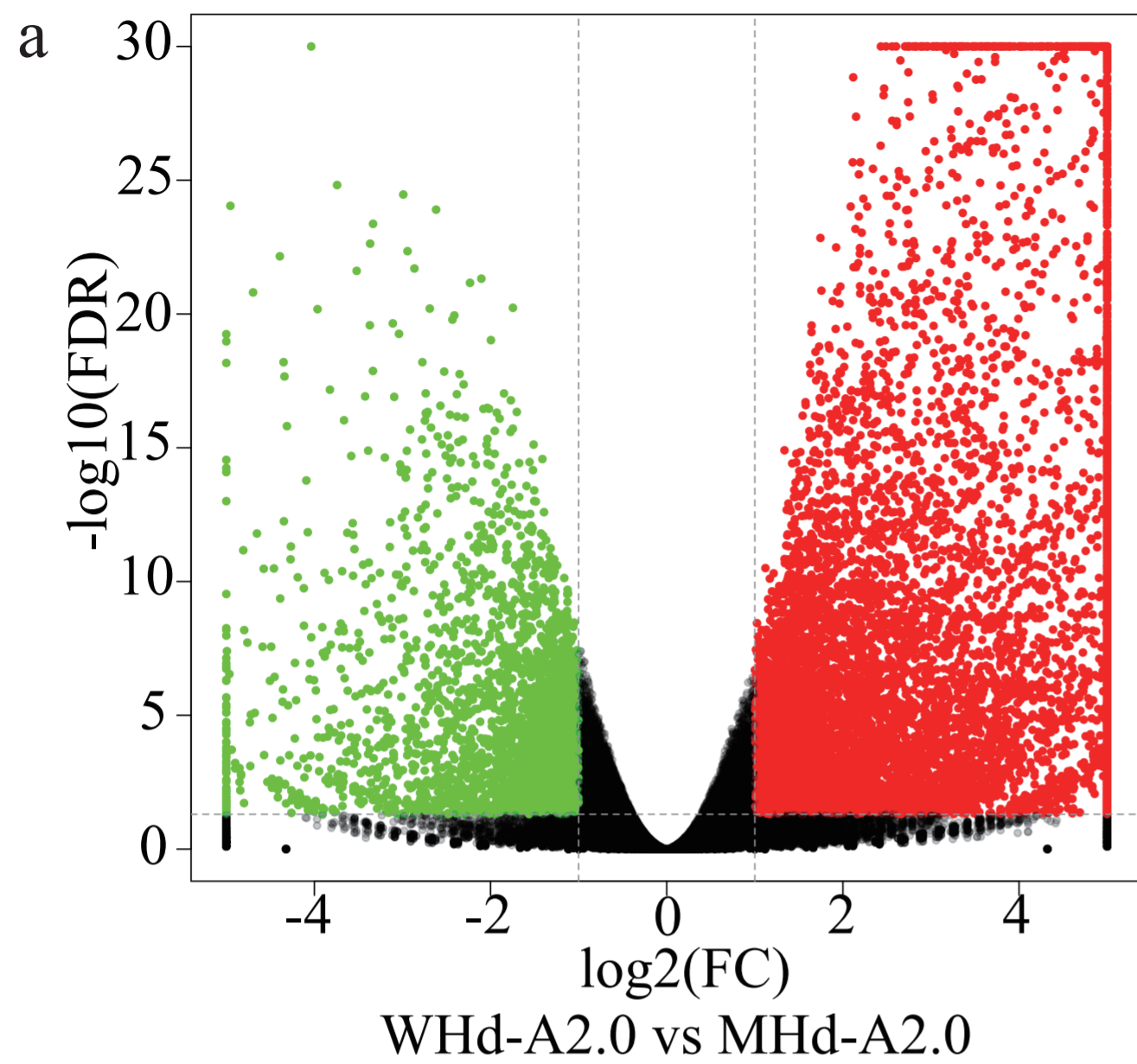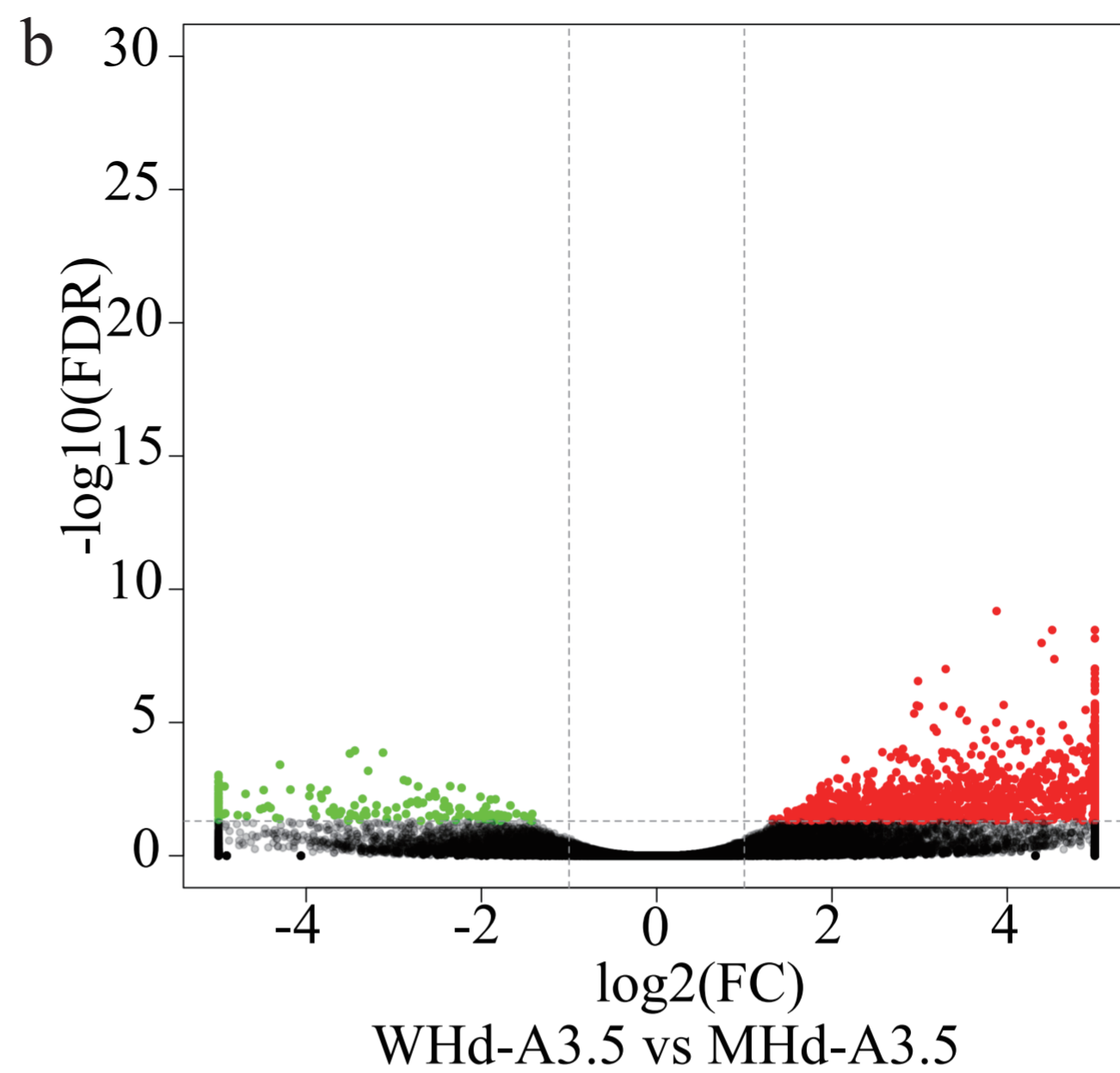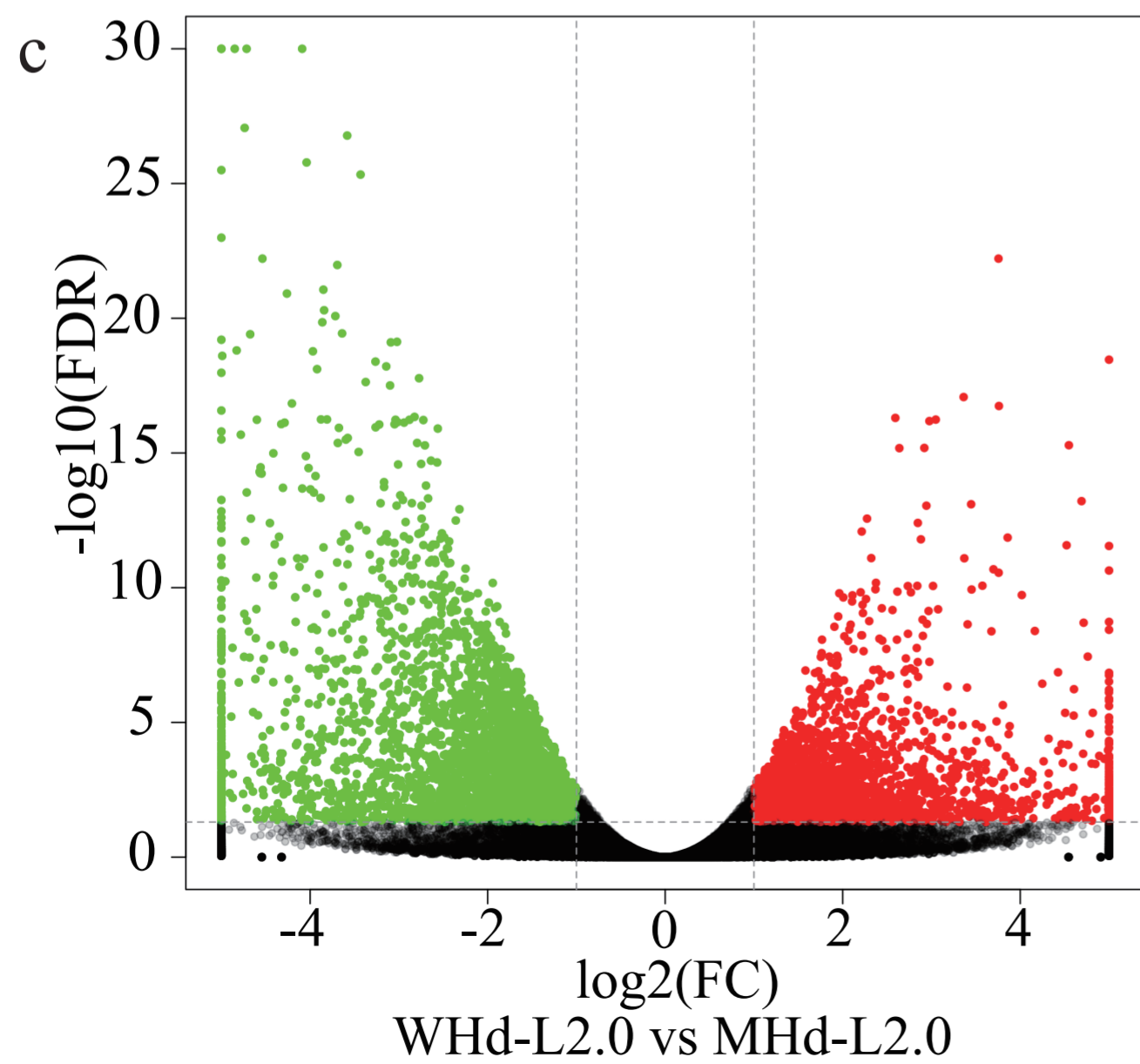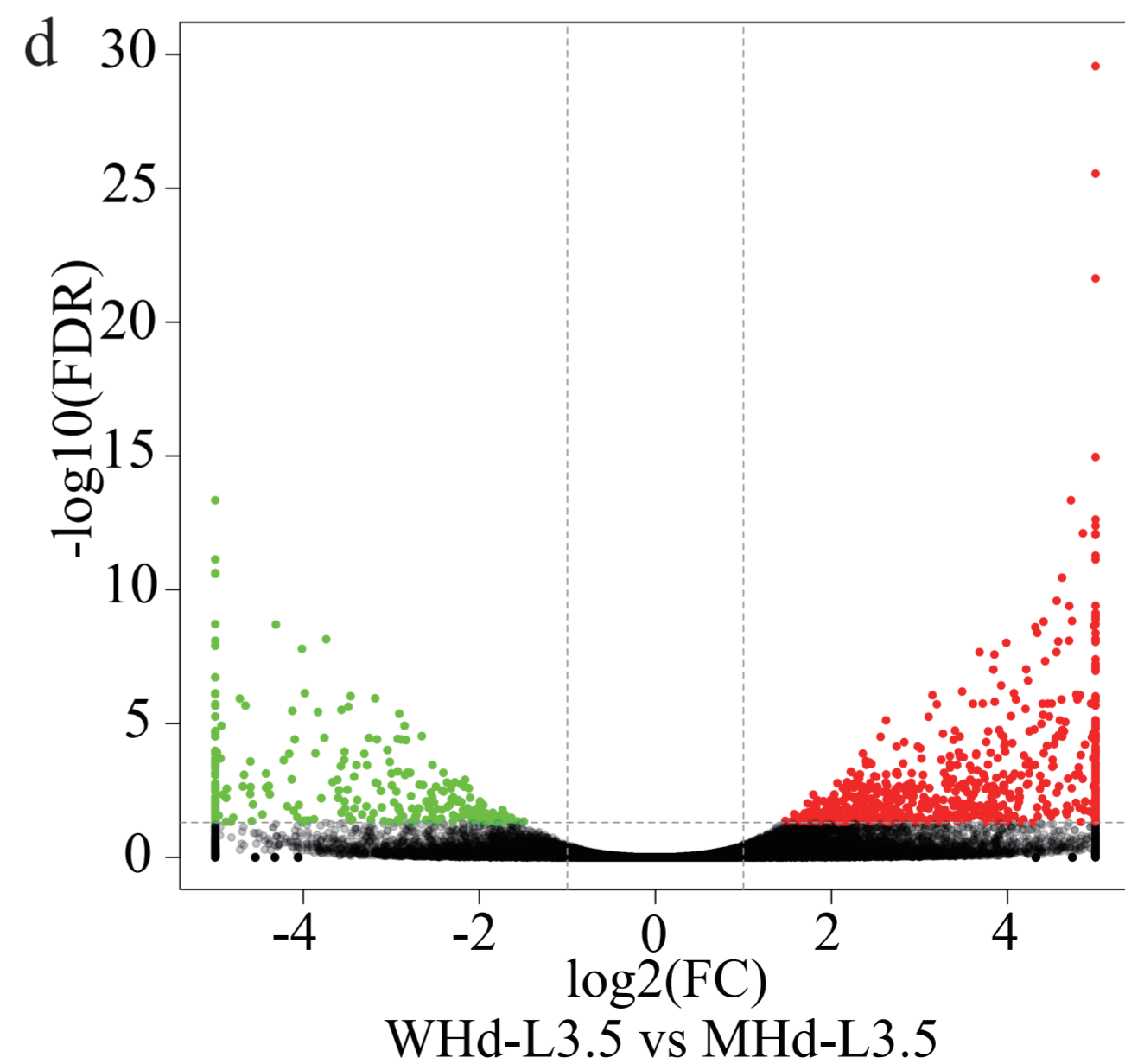

Supplement: Supplementary file 3 — Additional file 3: Figure S3. Volcano map of DEGs between WHd and MHd at different developmental stages and in different tissues. The abscissa represents the log2 (FC) value of each gene, and the ordinate corresponds to adjusted p-values. Green dots represent downregulated genes, red dots represent upregulated genes, and dark dots represent genes not differentially expressed. (a) Volcano map of DEGs between WHd-A2.0 vs MHd-A2.0. (b) Volcano map of DEGs between WHd-A3.5 vs MHd-A3.5. (c) Volcano map of DEGs between WHd-L2.0 vs MHd-L2.0. (d) Volcano map of DEGs between WHd-L3.5 vs MHd-L3.5. [file 12864_2021_7797_MOESM3_ESM.pdf]

Top 20 GO terms of WHd-A2.0 vs MHd-A2.0

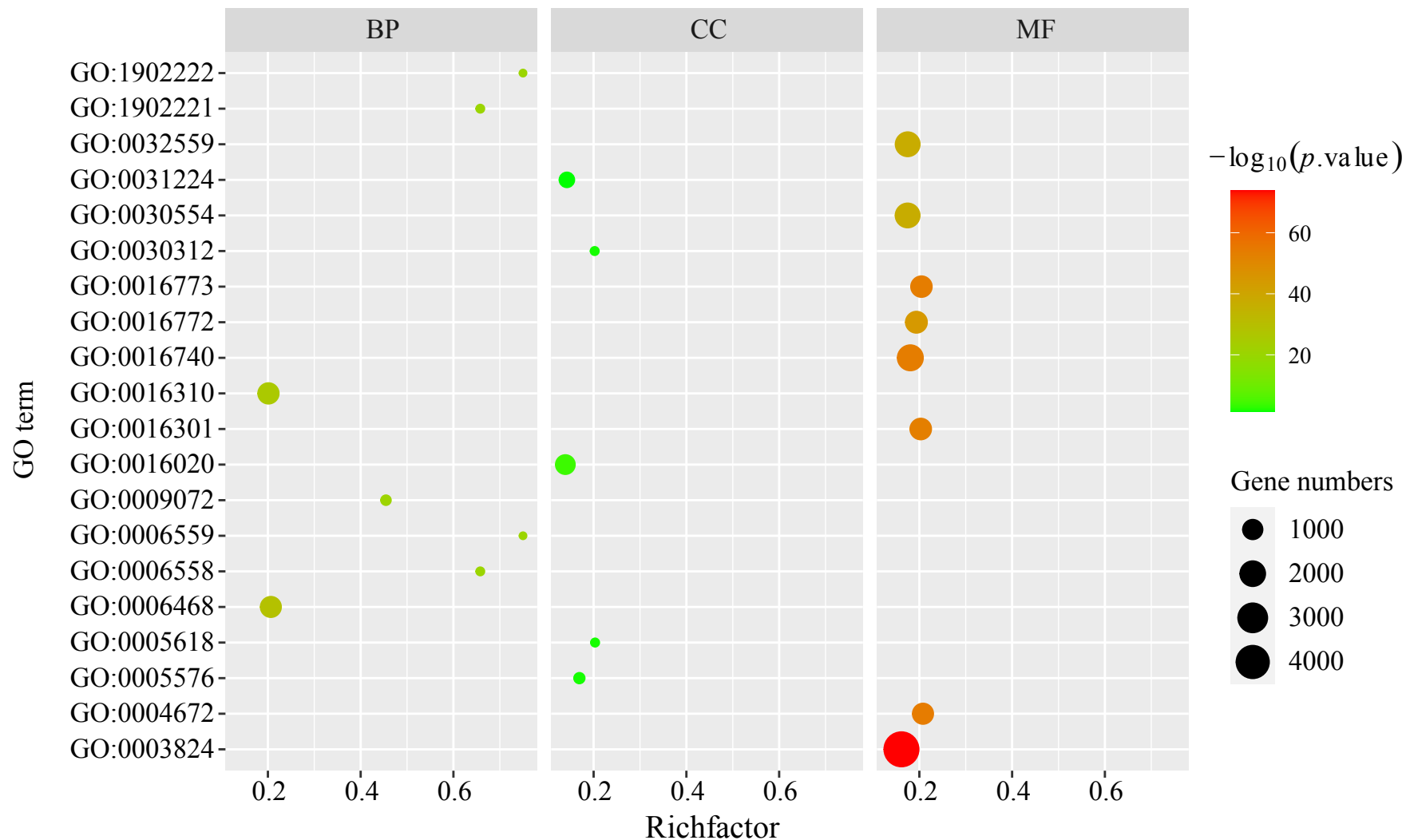

Supplement: Supplementary file 4 — Additional file 4: Figure S4. GO enrichment analysis of DEGs between WHd-A2.0 vs MHd-A2.0. [file 12864_2021_7797_MOESM4_ESM.pdf]

# Top 20 GO terms of WHd-L2.0 vs MHd-L2.0

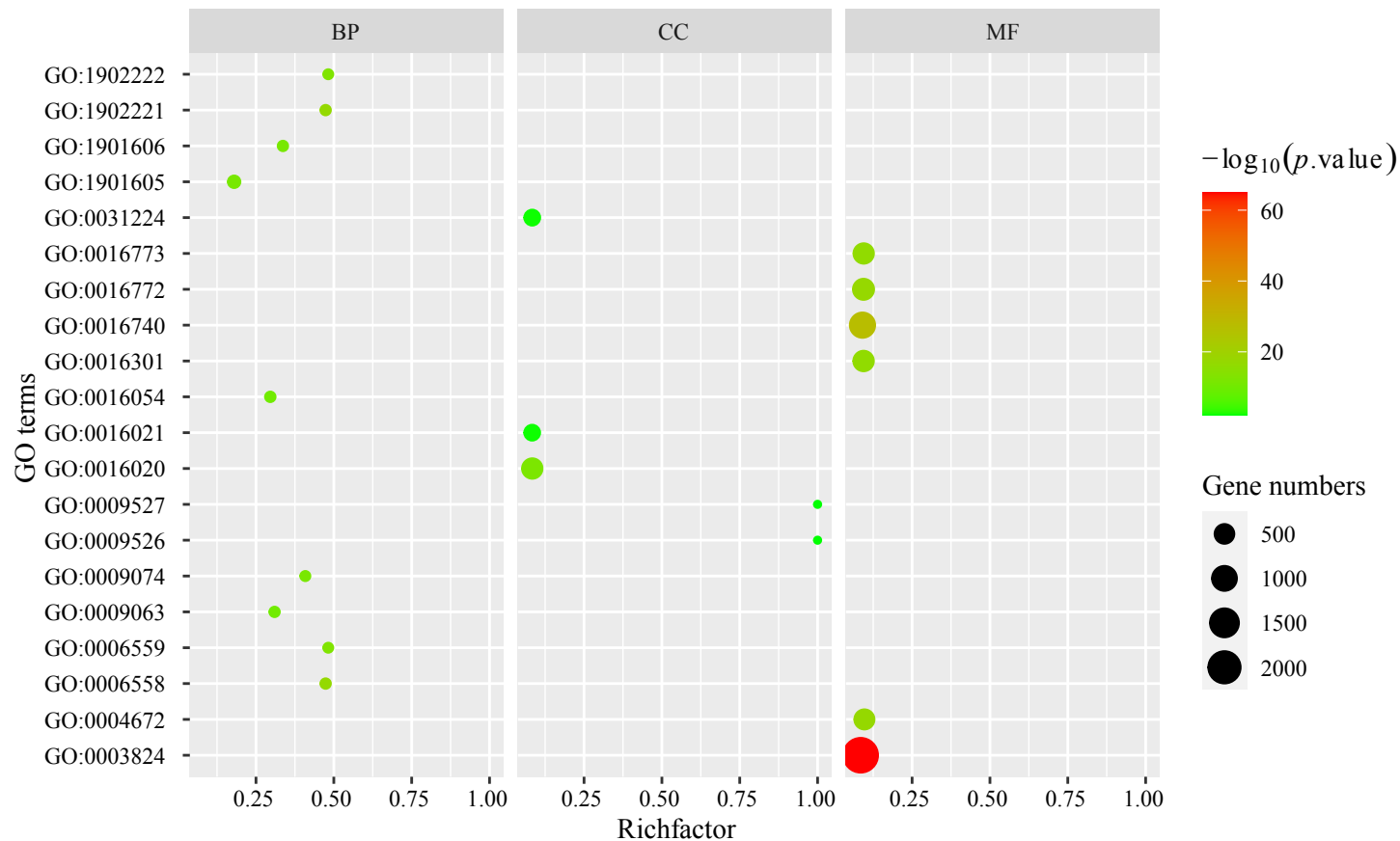

Supplement: Supplementary file 5 — Additional file 5: Figure S5. GO enrichment analysis of DEGs between WHd-L2.0 vs MHd.L2.0. [file 12864_2021_7797_MOESM5_ESM.pdf]

# Top 20 GO terms of WHd-L3.5 vs MHd-L3.5

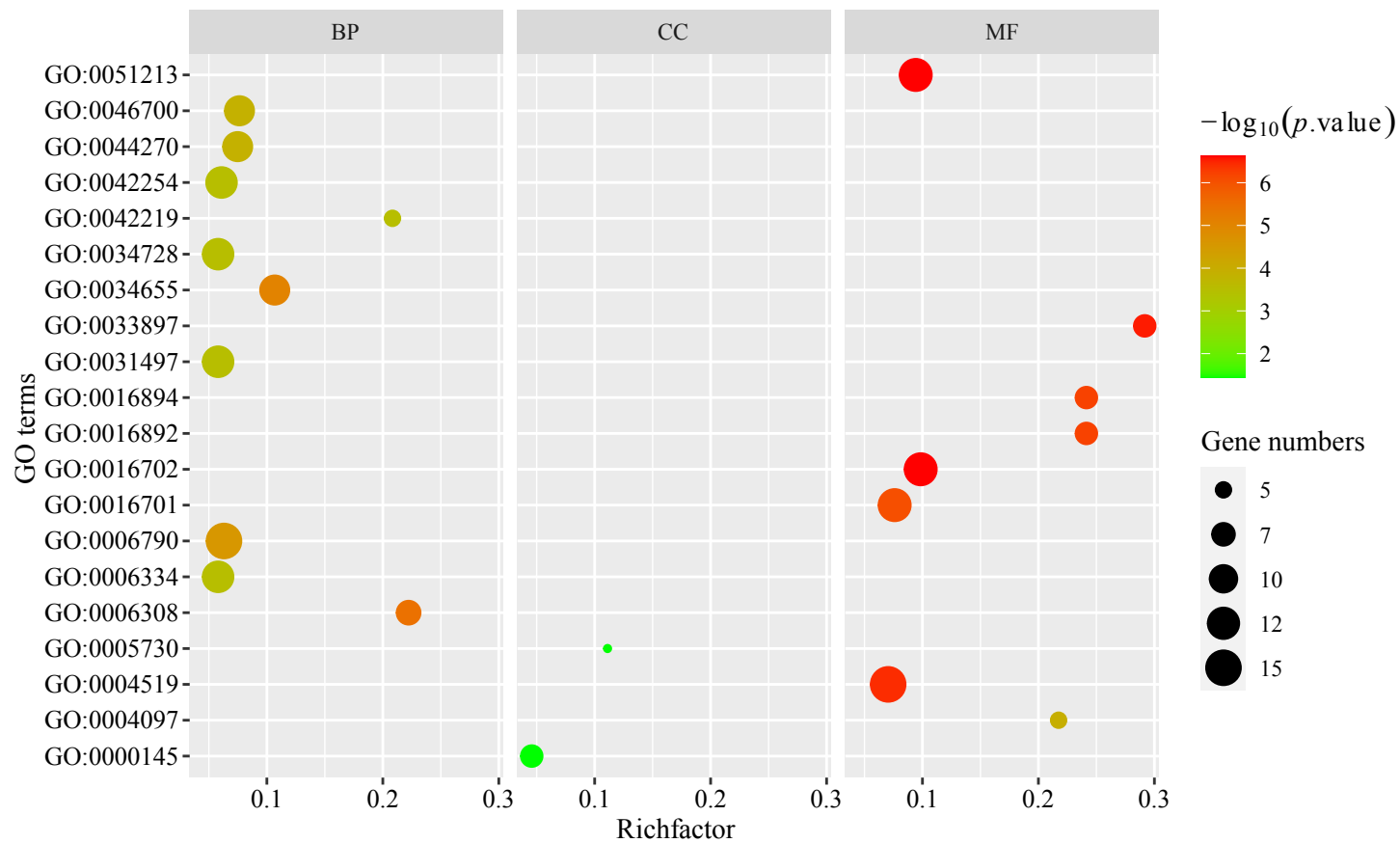

Supplement: Supplementary file 6 — Additional file 6: Figure S6. GO enrichment analysis of DEGs between WHd-L3.5 vs MHd-L3.5. [file 12864_2021_7797_MOESM6_ESM.pdf]

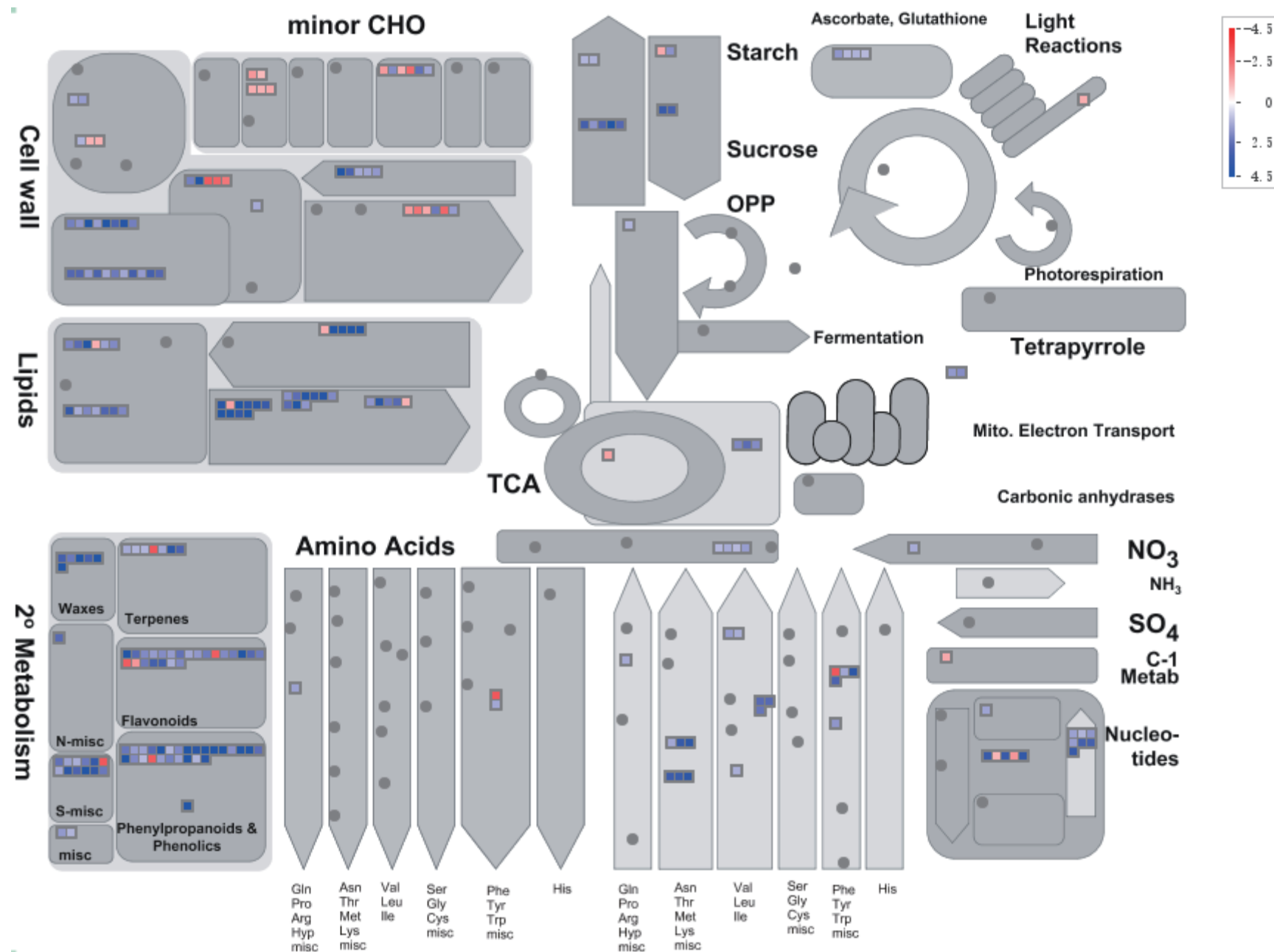

Supplement: Supplementary file 7 — Additional file 7: Figure S7. Overview of the metabolic pathway of differentially expressed genes between WHd-A2.0 vs MHd-A2.0. [file 12864_2021_7797_MOESM7_ESM.pdf]

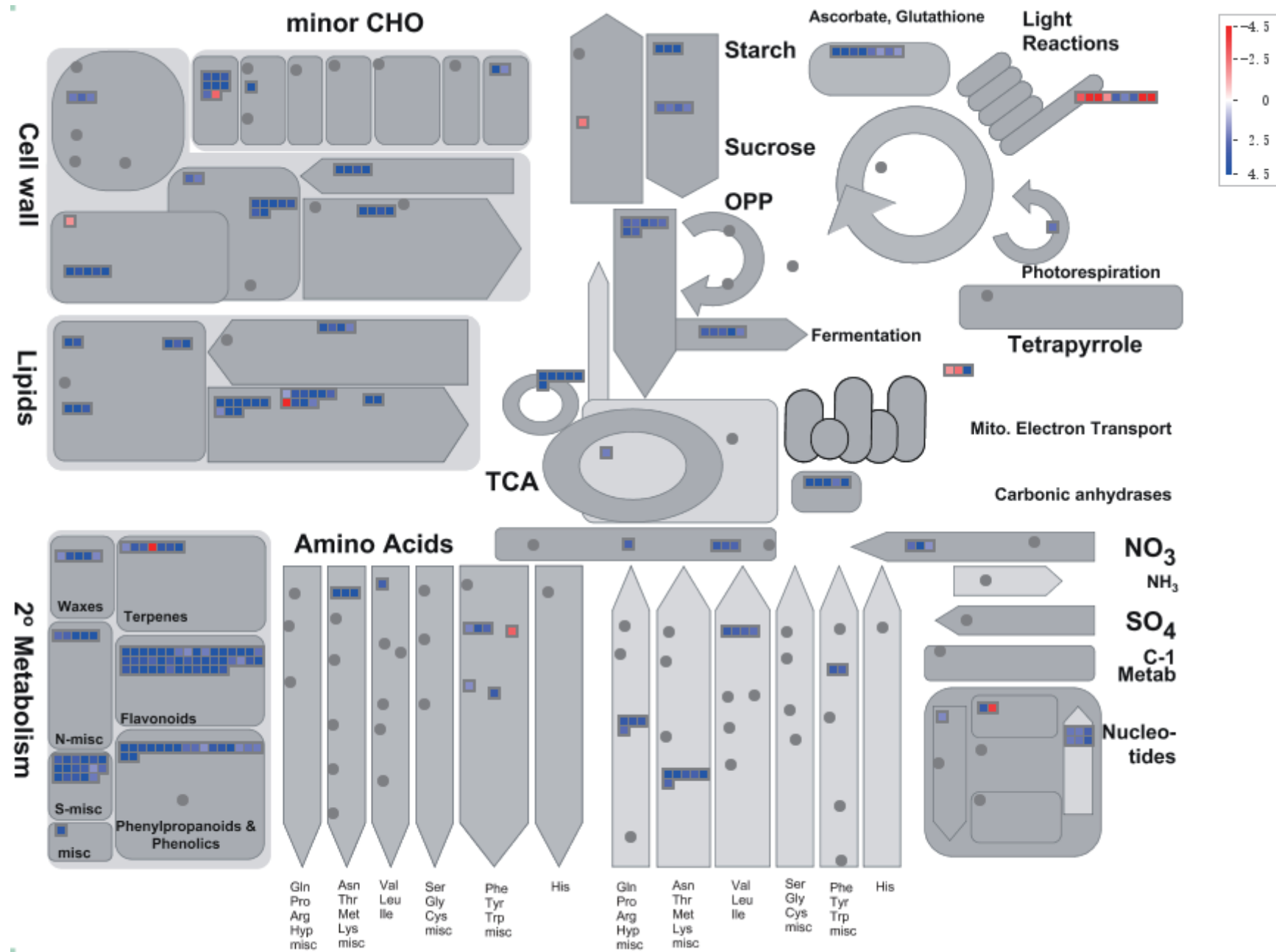

Supplement: Supplementary file 8 — Additional file 8: Figure S8. Overview of the metabolic pathway of differentially expressed genes between WHd-A3.5 vs MHd-A3.5. [file 12864_2021_7797_MOESM8_ESM.pdf]

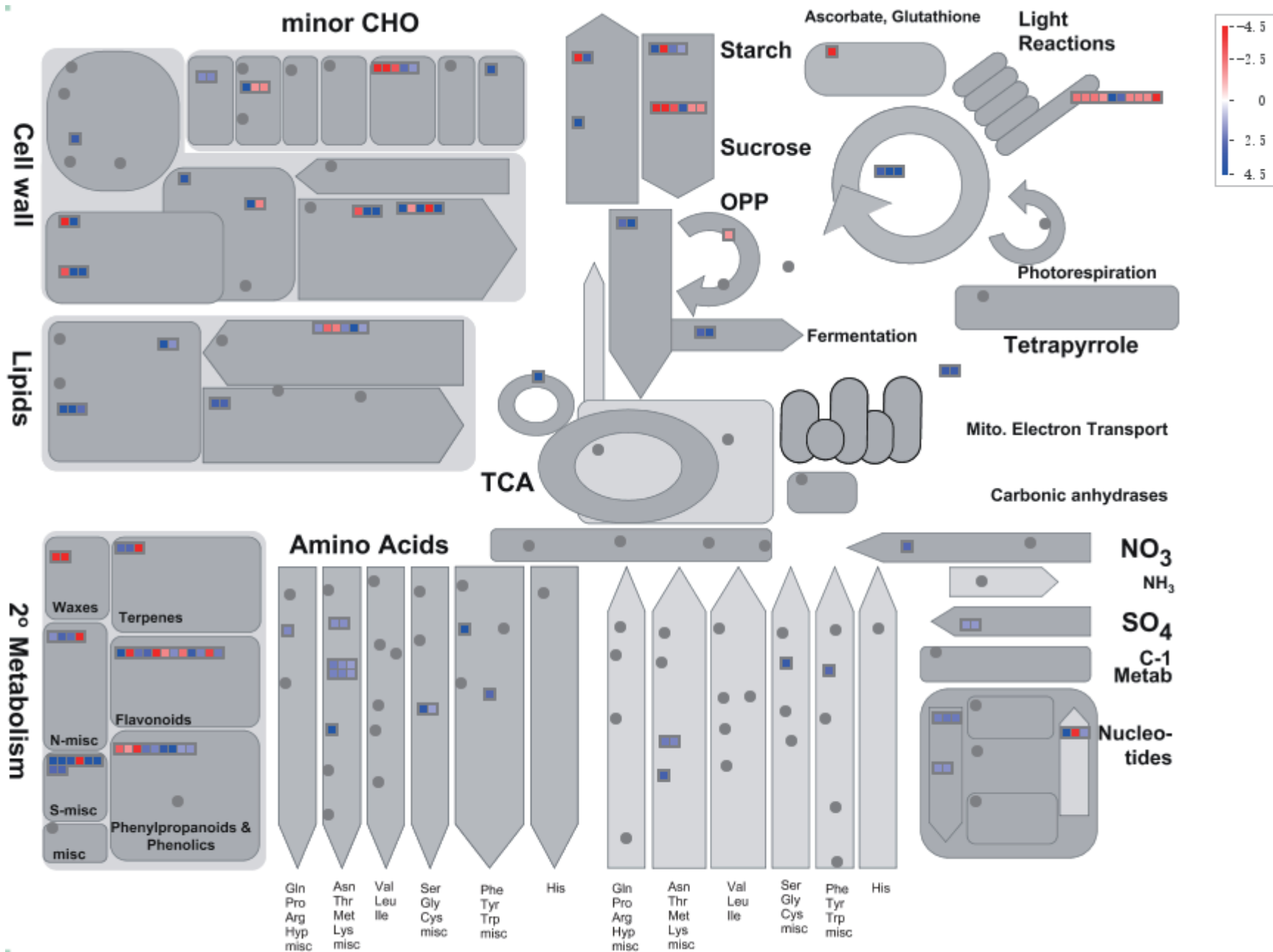

Supplement: Supplementary file 9 — Additional file 9: Figure S9. Overview of the metabolic pathway of differentially expressed genes between WHd-L3.5 vs MHd-L3.5. [file 12864_2021_7797_MOESM9_ESM.pdf]

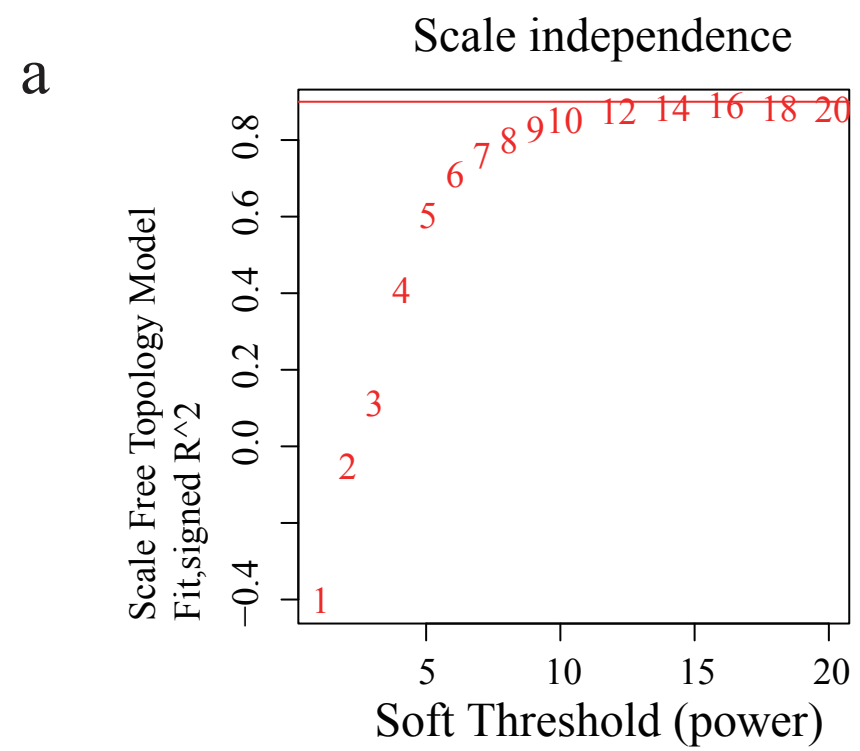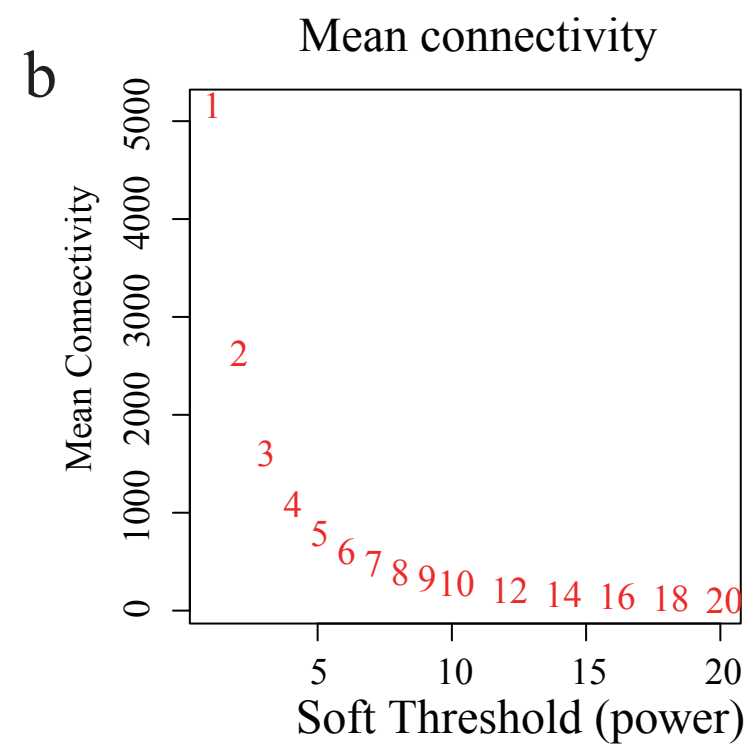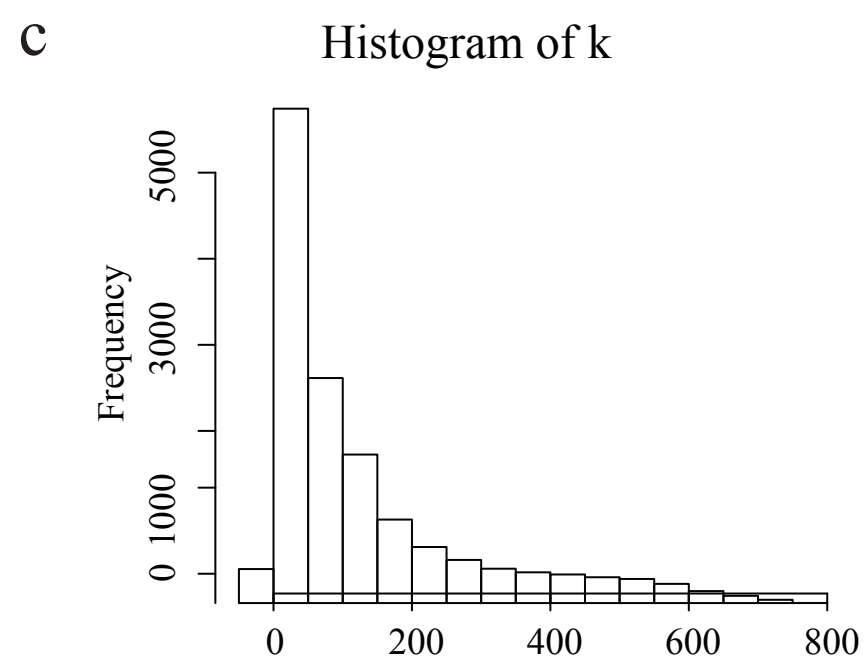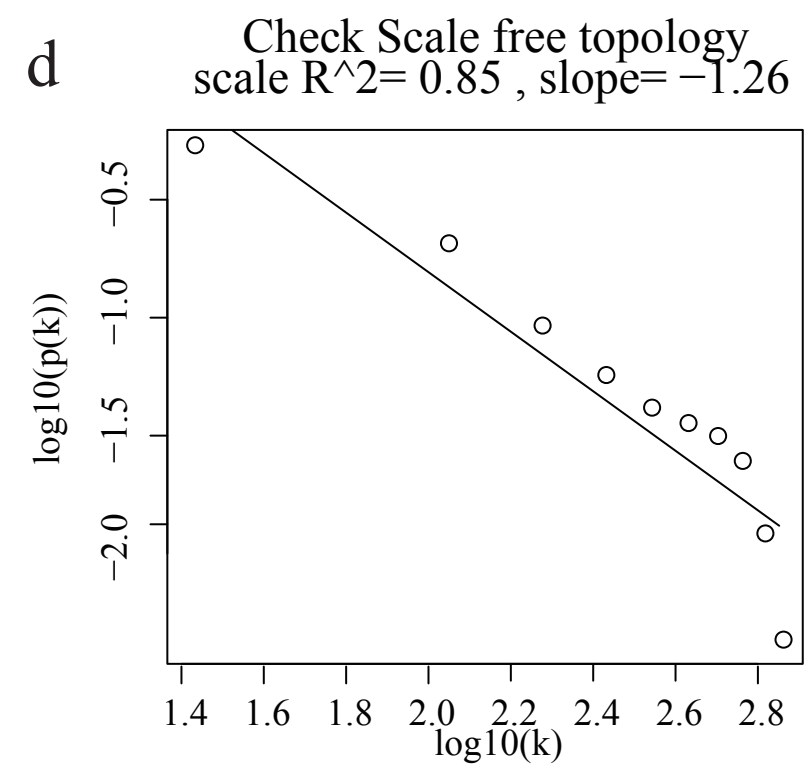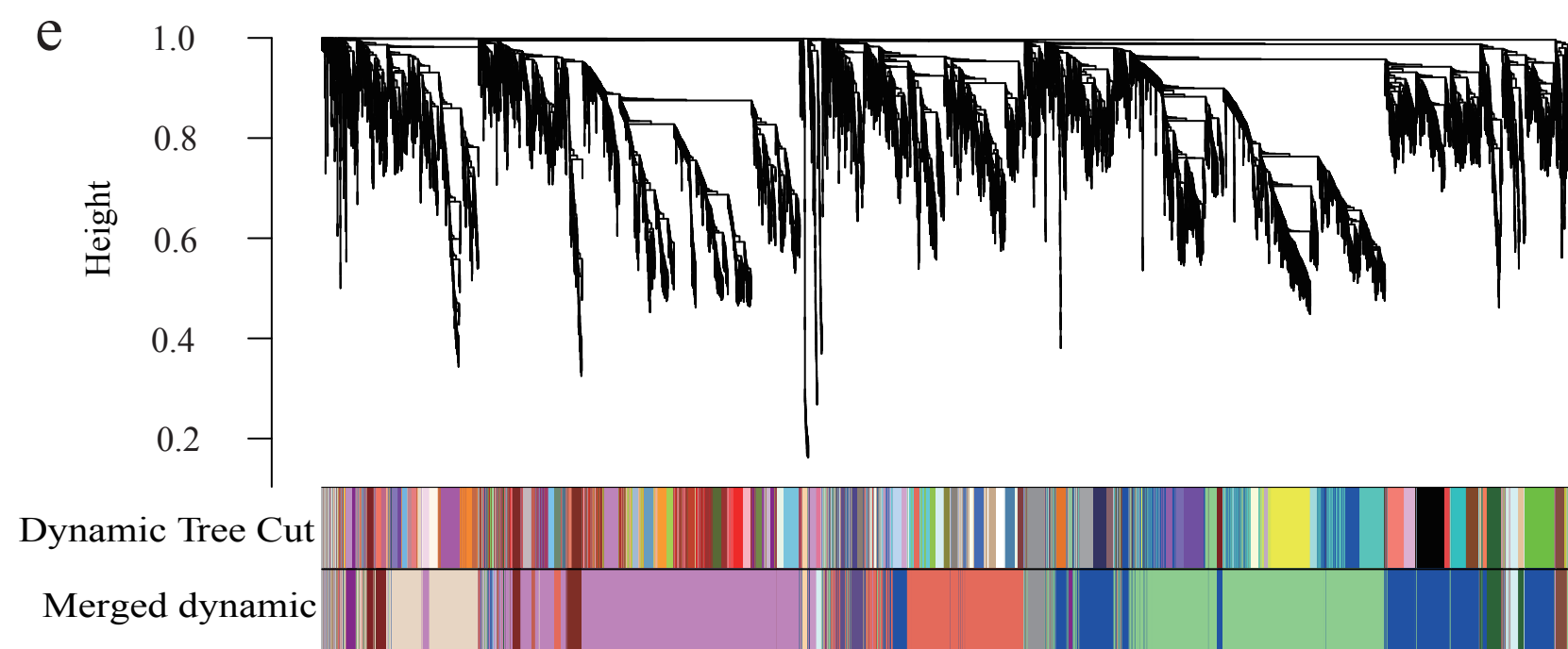

Supplement: Supplementary file 10 — Additional file 10: Figure S10. Identification of the soft-threshold power in WGCNA. (a) Scale-free fit index analysis under different soft-thresholding powers (β). Ordinates represent the relationship between the degree of connection (k) and P(k). (b) Mean gene connectivity analysis under different soft-thresholding powers (β). (c) Histogram of connectivity distribution when β = 14. (d) A check of the scale-free topology when β = 14. (e) Dendrogram of all differentially expressed genes clustered based on a dissimilarity measure (1-TOM). [file 12864_2021_7797_MOESM10_ESM.pdf]

Module–Trait Relationships

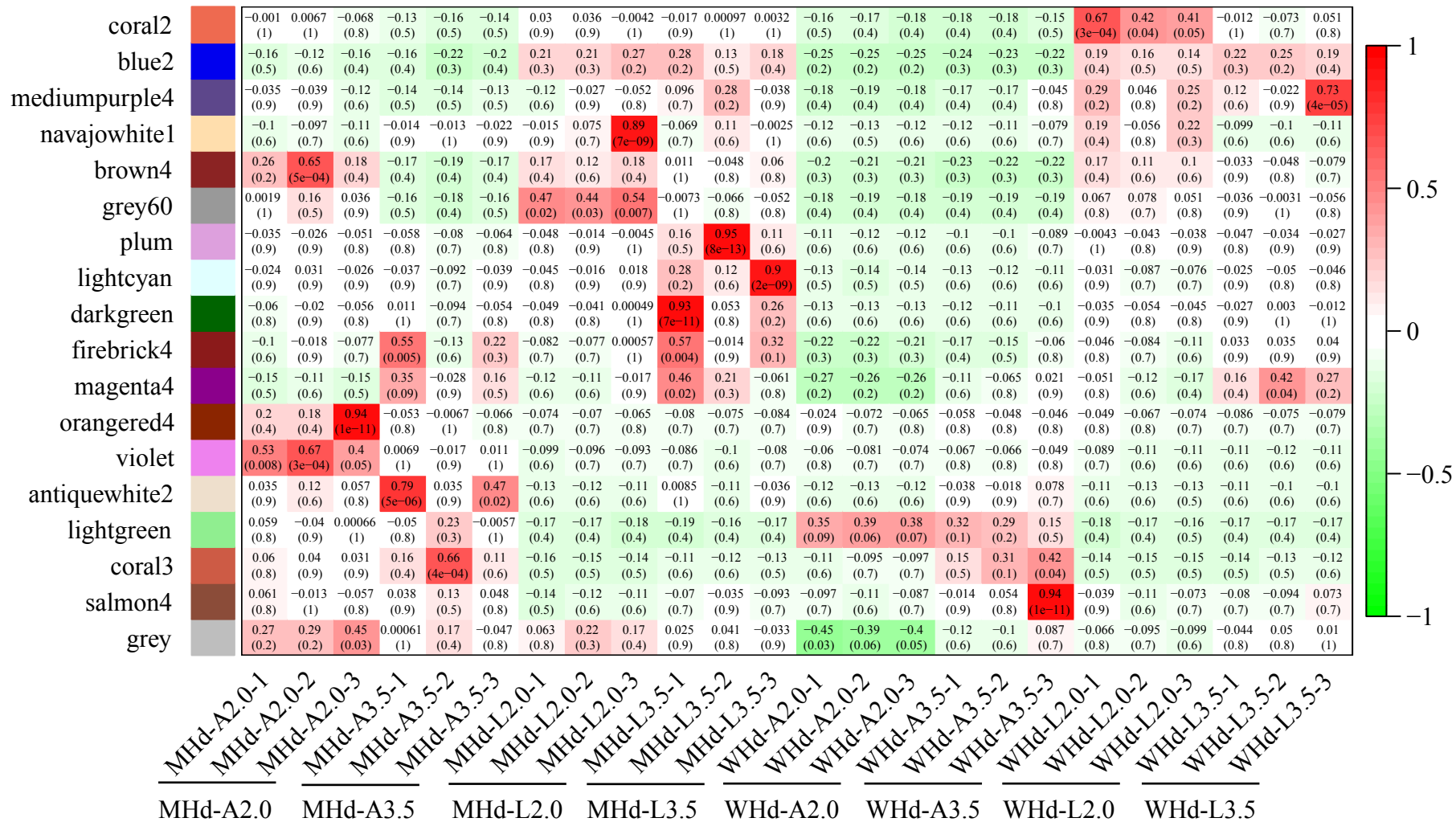

Supplement: Supplementary file 11 — Additional file 11: Figure S11. Heatmap of module-trait relationships. Each row is a module eigenvalue that is represented by different colors, and each column represents a sample name. Correlation between sample names and module eigenvalues was calculated by the Pearson correlation coefficient. Red represents a high correlation and green represents a negative correlation between samples and module eigenvalues. [file 12864_2021_7797_MOESM11_ESM.pdf]

a

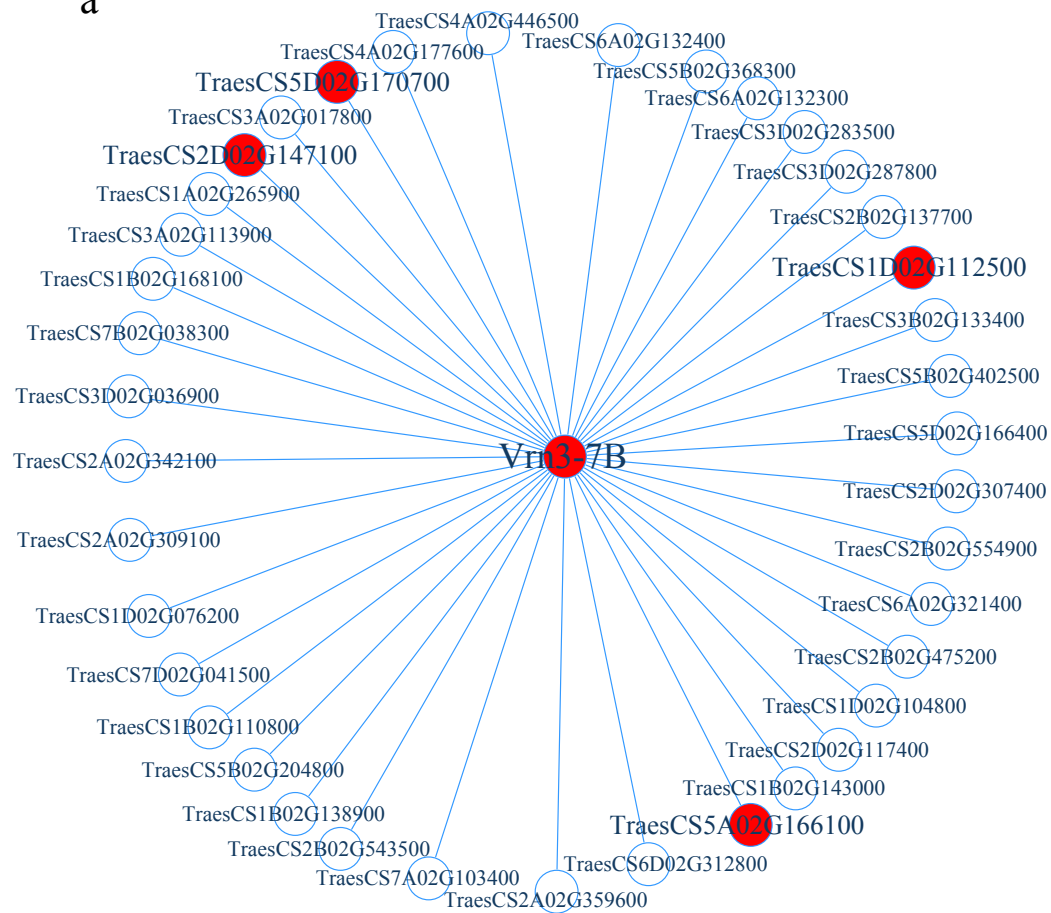

b

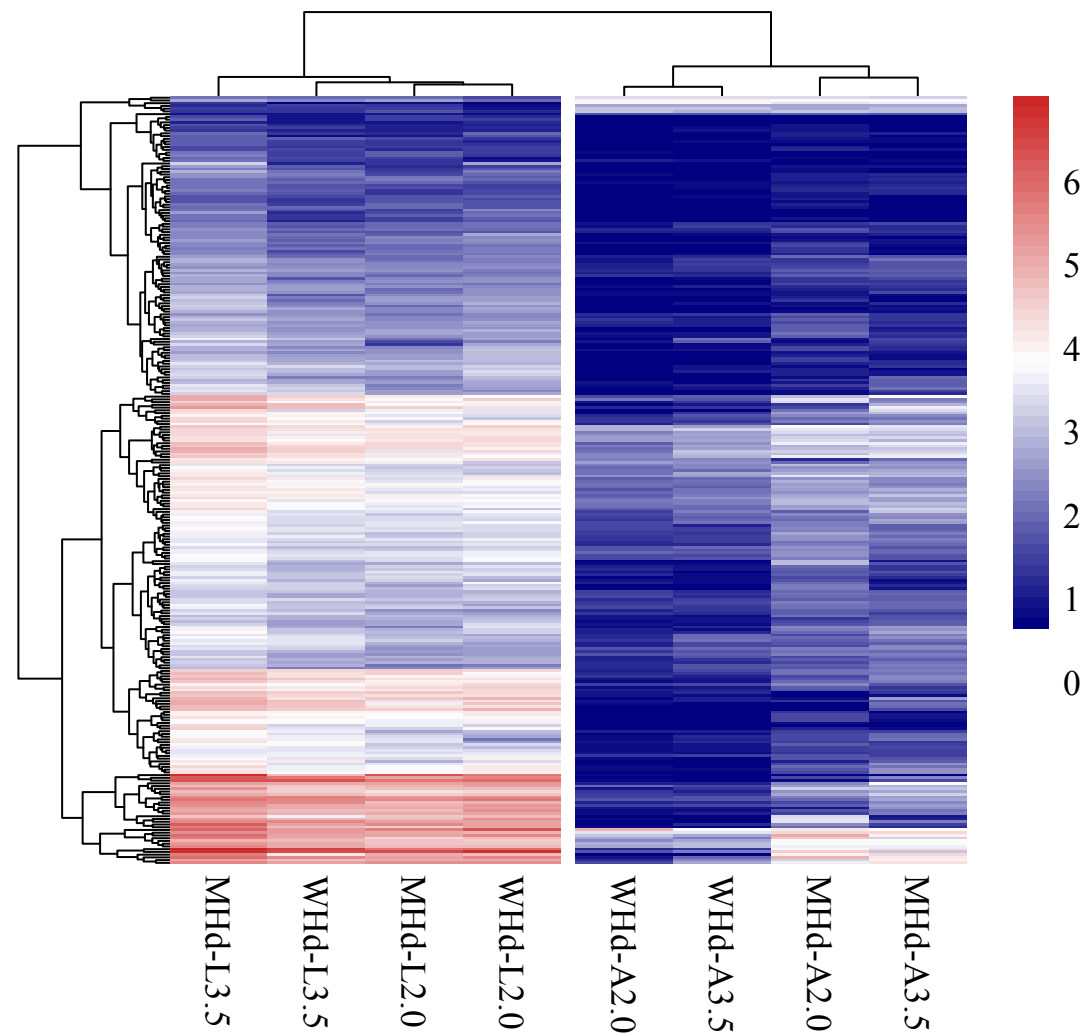

Supplement: Supplementary file 12 — Additional file 12: Figure S12. Construction of the flowering time regulatory network and expression level of the differential genes. The red nodes in the network indicate high-confidence genes involved in the heading date. (a) The Vrn3-7B flowering time regulatory network. (b) The expression heatmap of differentially expressed genes that were co-expressed with Vrn3-7B. [file 12864_2021_7797_MOESM12_ESM.pdf]

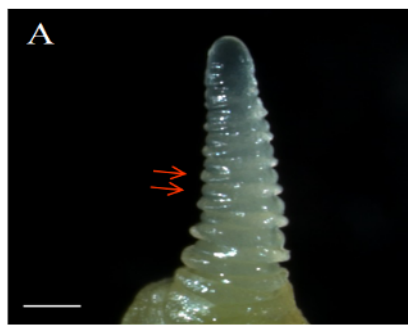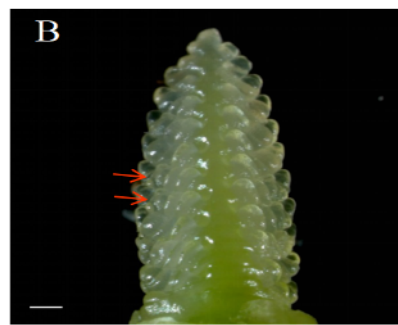

bar=200um

Supplement: Supplementary file 15 — Additional file 15: Figure S15. The development stages of the shoot apex. A: Double ridge stage; B: Pistil and stamen primordium differentiation stage. Note: The red arrow indicates the characteristics of the development period. [file 12864_2021_7797_MOESM15_ESM.pdf]
