# Supplementary material for: Transcriptome profiling of developing leaf and shoot apices to reveal the molecular mechanism and co-expression genes responsible for the wheat heading date
Source: BMC Genomics. 2021 Jun 23;22:468. doi: 10.1186/s12864-021-07797-7 (PMC8220847; doi:10.1186/s12864-021-07797-7)
Supplement: Supplementary file 13 — Additional file 13: Figure S13. Construction of the flowering time regulatory network and expression level of differential genes. The red nodes in the network indicate high-confidence genes involved in the heading date. (a) The Ppd1-1D flowering time regulatory network. (b) The expression heatmap of differentially expressed genes co-expressed with Ppd1-1D. [file 12864_2021_7797_MOESM13_ESM.pdf]

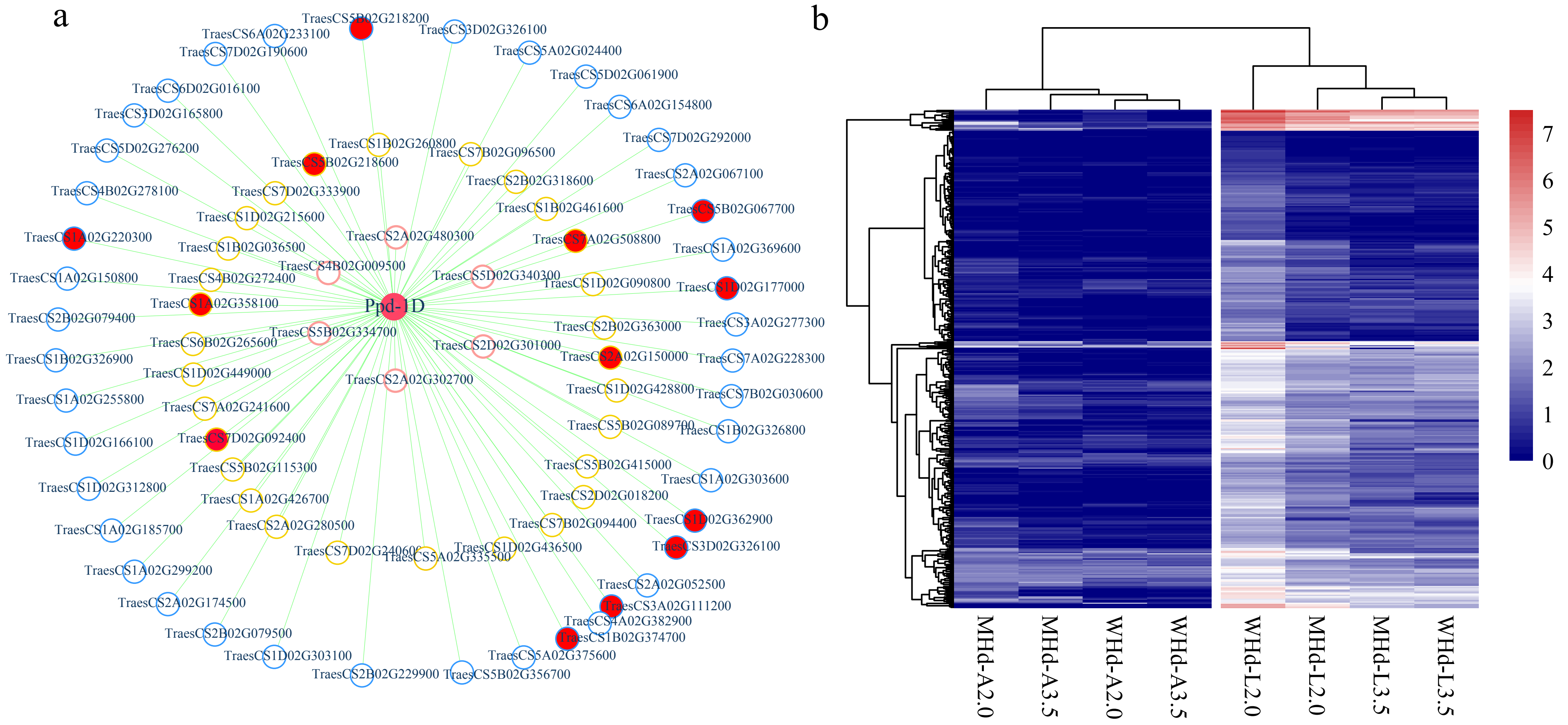

a
TraesCS5B02G218200
TraesCS6A02G233100
TraesCS3D02G326100
TraesCS7D02G190600
TraesCS5A02G024400
TraesCS5D02G061900
TraesCS6D02G016100
TraesCS6A02G154800
TraesCS3D02G165800
TraesCS7D02G292000
TraesCS1B02G260800
TraesCS5D02G276200
TraesCS5B02G218600
TraesCS7B02G096500
TraesCS2A02G067100
TraesCS2B02G318600
TraesCS4B02G278100
TraesCS7D02G333900
TraesCS1B02G461600
TraesCS5B02G067700
TraesCS1D02G215600
TraesCS2A02G480300
TraesCS1A02G220300
TraesCS7A02G508800
TraesCS1B02G036500
TraesCS1A02G369600
TraesCS4B02G009500
TraesCS1A02G150800
TraesCS4B02G272400
TraesCS5D02G340300
TraesCS1D02G090800
TraesCS1D02G177000
TraesCS1A02G358100
Ppd-1D
TraesCS2B02G079400
TraesCS2B02G363000
TraesCS3A02G277300
TraesCS5B02G334700
TraesCS6B02G265600
TraesCS2D02G301000
TraesCS2A02G150000
TraesCS7A02G228300
TraesCS1B02G326900
TraesCS1D02G449000
TraesCS2A02G302700
TraesCS1D02G428800
TraesCS7B02G030600
TraesCS1A02G255800
TraesCS7A02G241600
TraesCS5B02G089700
TraesCS1B02G326800
TraesCS1D02G166100
TraesCS7D02G092400
TraesCS5B02G415000
TraesCS5B02G115300
TraesCS1A02G303600
TraesCS1D02G312800
TraesCS2D02G018200
TraesCS1A02G426700
TraesCS1D02G362900
TraesCS7B02G094400
TraesCS1A02G185700
TraesCS2A02G280500
TraesCS3D02G326100
TraesCS1D02G436500
TraesCS5A02G335500
TraesCS1A02G299200
TraesCS2A02G052500
TraesCS2A02G174500
TraesCS3A02G111200
TraesCS4A02G382900
TraesCS2B02G079500
TraesCS1B02G374700
TraesCS1D02G303100
TraesCS5A02G375600
TraesCS2B02G229900
TraesCS5B02G356700
b
7
6
5
4
3
2
1
0
MHd-A2.0
MHd-A3.5
WHd-A2.0
WHd-A3.5
WHd-L2.0
MHd-L2.0
MHd-L3.5
WHd-L3.5
