# Supplementary material for: Transcriptome profiling of developing leaf and shoot apices to reveal the molecular mechanism and co-expression genes responsible for the wheat heading date
Source: BMC Genomics. 2021 Jun 23;22:468. doi: 10.1186/s12864-021-07797-7 (PMC8220847; doi:10.1186/s12864-021-07797-7)
Supplement: Supplementary file 14 — Additional file 14: Figure S14. Construction of the flowering time regulatory network and expression level of the differential genes. The red nodes in the network indicate high-confidence genes involved in the heading date. (a) Representative WSOC1 flowering time regulatory network. (b) The expression heatmap of differentially expressed genes that were co-expressed with WSOC1. [file 12864_2021_7797_MOESM14_ESM.pdf]

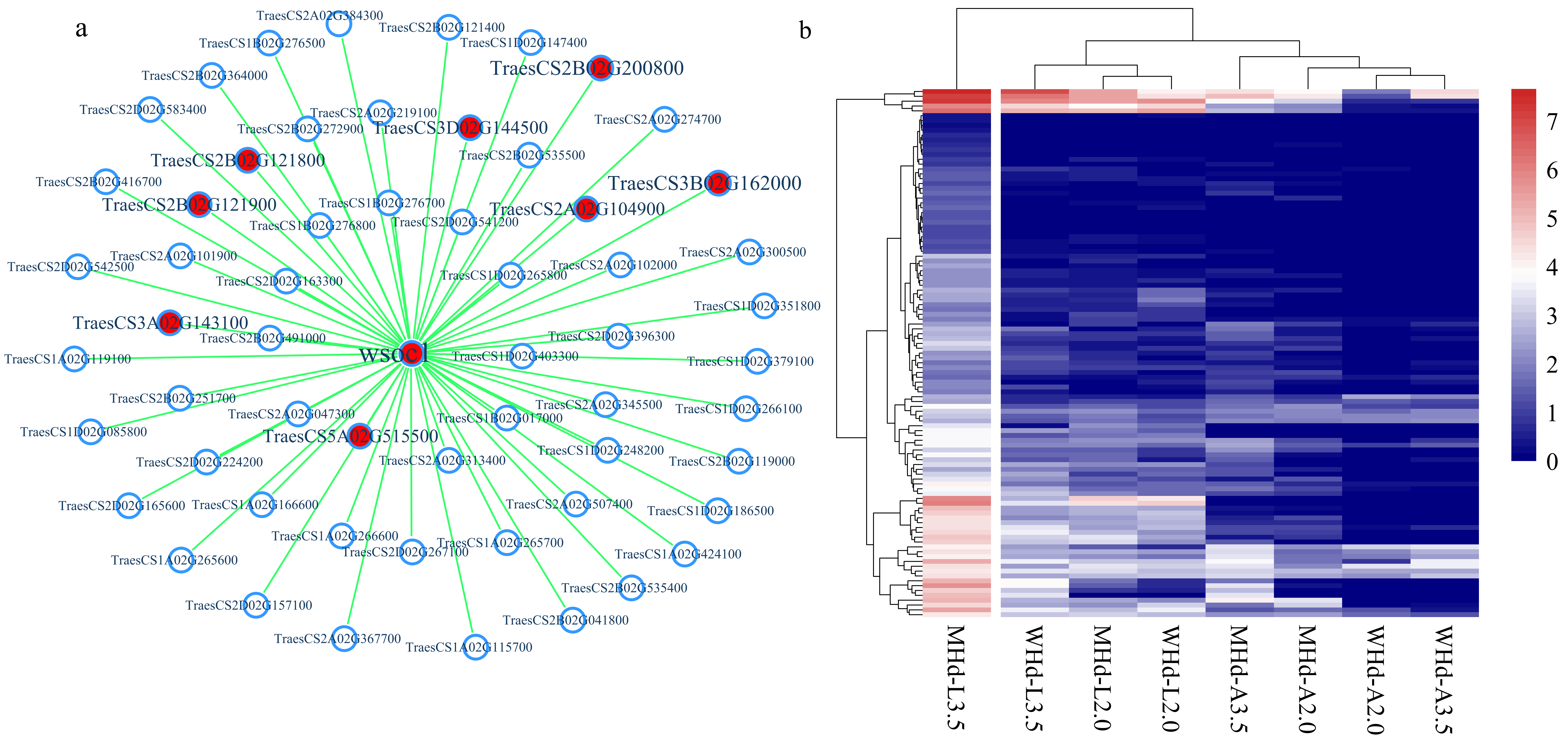
a
TraesCS2A02G384300
TraesCS2B02G121400
TraesCS1B02G276500
TraesCS1D02G147400
TraesCS2B02G200800
TraesCS2B02G364000
TraesCS2D02G583400
TraesCS2A02G219100
TraesCS2B02G272900
TraesCS3D02G144500
TraesCS2A02G274700
TraesCS2B02G121800
TraesCS2B02G535500
TraesCS2B02G416700
TraesCS3B02G162000
TraesCS2B02G121900
TraesCS1B02G276700
TraesCS2A02G104900
TraesCS1B02G276800
TraesCS2D02G541200
TraesCS2A02G101900
TraesCS2D02G542500
TraesCS2A02G300500
TraesCS2D02G163300
TraesCS1D02G265800
TraesCS2A02G102000
TraesCS1D02G351800
TraesCS3A02G143100
TraesCS2D02G396300
TraesCS2B02G491000
TraesCS1A02G119100
WSOC1
TraesCS1D02G403300
TraesCS1D02G379100
TraesCS2B02G251700
TraesCS2A02G345500
TraesCS1D02G266100
TraesCS2A02G047300
TraesCS1B02G017000
TraesCS1D02G085800
TraesCS5A02G515500
TraesCS1D02G248200
TraesCS2D02G224200
TraesCS2A02G313400
TraesCS2B02G119000
TraesCS2D02G165600
TraesCS1A02G166600
TraesCS2A02G507400
TraesCS1D02G186500
TraesCS1A02G266600
TraesCS1A02G265700
TraesCS2D02G267100
TraesCS1A02G424100
TraesCS1A02G265600
TraesCS2B02G535400
TraesCS2D02G157100
TraesCS2B02G041800
TraesCS2A02G367700
TraesCS1A02G115700
b
7
6
5
4
3
2
1
0
MHd-L3.5
WHd-L3.5
MHd-L2.0
WHd-L2.0
MHd-A3.5
MHd-A2.0
WHd-A2.0
WHd-A3.5
